# Four-pronged negative feedback of DSB machinery in meiotic DNA-break control in mice

**DOI:** 10.1093/nar/gkab082

**Published:** 2021-02-22

**Authors:** Ihsan Dereli, Marcello Stanzione, Fabrizio Olmeda, Frantzeskos Papanikos, Marek Baumann, Sevgican Demir, Fabrizia Carofiglio, Julian Lange, Bernard de Massy, Willy M Baarends, James Turner, Steffen Rulands, Attila Tóth

**Affiliations:** Institute of Physiological Chemistry, Faculty of Medicine at the TU Dresden, Fiedlerstrasse 42, 01307 Dresden, Germany; Institute of Physiological Chemistry, Faculty of Medicine at the TU Dresden, Fiedlerstrasse 42, 01307 Dresden, Germany; Max Planck Institute for the Physics of Complex Systems, Noethnitzer Strasse 38, 01187 Dresden, Germany; Institute of Physiological Chemistry, Faculty of Medicine at the TU Dresden, Fiedlerstrasse 42, 01307 Dresden, Germany; Institute of Physiological Chemistry, Faculty of Medicine at the TU Dresden, Fiedlerstrasse 42, 01307 Dresden, Germany; Institute of Physiological Chemistry, Faculty of Medicine at the TU Dresden, Fiedlerstrasse 42, 01307 Dresden, Germany; Department of Developmental Biology, Erasmus MC - University Medical Center, Rotterdam, The Netherlands; Molecular Biology Program, Memorial Sloan Kettering Cancer Center, New York, NY 10065, USA; Howard Hughes Medical Institute, Memorial Sloan Kettering Cancer Center, New York, NY 10065, USA; Institute of Human Genetics, UMR 9002, CNRS, Université de Montpellier, 34396 Montpellier cedex 5, France; Department of Developmental Biology, Erasmus MC - University Medical Center, Rotterdam, The Netherlands; Sex Chromosome Biology Laboratory, The Francis Crick Institute, London, NW1 1AT, UK; Max Planck Institute for the Physics of Complex Systems, Noethnitzer Strasse 38, 01187 Dresden, Germany; Center for Systems Biology Dresden (CSBD), Pfotenhauer Strasse 108, 01307 Dresden, Germany; Institute of Physiological Chemistry, Faculty of Medicine at the TU Dresden, Fiedlerstrasse 42, 01307 Dresden, Germany

## Abstract

In most taxa, halving of chromosome numbers during meiosis requires that homologous chromosomes (homologues) pair and form crossovers. Crossovers emerge from the recombination-mediated repair of programmed DNA double-strand breaks (DSBs). DSBs are generated by SPO11, whose activity requires auxiliary protein complexes, called pre-DSB recombinosomes. To elucidate the spatiotemporal control of the DSB machinery, we focused on an essential SPO11 auxiliary protein, IHO1, which serves as the main anchor for pre-DSB recombinosomes on chromosome cores, called axes. We discovered that DSBs restrict the DSB machinery by at least four distinct pathways in mice. Firstly, by activating the DNA damage response (DDR) kinase ATM, DSBs restrict pre-DSB recombinosome numbers without affecting IHO1. Secondly, in their vicinity, DSBs trigger IHO1 depletion mainly by another DDR kinase, ATR. Thirdly, DSBs enable homologue synapsis, which promotes the depletion of IHO1 and pre-DSB recombinosomes from synapsed axes. Finally, DSBs and three DDR kinases, ATM, ATR and PRKDC, enable stage-specific depletion of IHO1 from all axes. We hypothesize that these four negative feedback pathways protect genome integrity by ensuring that DSBs form without excess, are well-distributed, and are restricted to genomic locations and prophase stages where DSBs are functional for promoting homologue pairing and crossover formation.

## INTRODUCTION

Chromosome numbers are halved in germ cells during meiosis as a single round of pre-meiotic DNA replication is followed by two rounds of chromosome segregations. Orderly chromosome segregation in the first meiotic division mechanistically requires conjoining of homologous copies (homologues) of each chromosome by the formation of physical linkages in the first meiotic prophase (reviewed in (1)). In most taxa, physical linkages between homologues rely on reciprocal inter-homologue DNA exchanges, called crossovers. Meiotic homologous recombination generates crossovers by the repair of programmed DNA double-stranded breaks (DSBs), whose induction at the start of meiosis depends on the topoisomerase-like enzyme, SPO11, and its co-factor TOPOVIBl (2–7). DSBs both constitute a potentially toxic DNA damage and enable meiotic recombination, hence DSB formation is under tight spatiotemporal control in meiosis (8–12).

Chromatin is organized into DNA loops that emanate from linear proteinaceous chromosome cores, called chromosome axes, in meiosis (13). DSBs form in association with chromosome axes. To generate DSBs SPO11 requires numerous auxiliary pro-DSB proteins (reviewed in (12,14)). In mammals, at least five pro-DSB proteins—MEI4, REC114, IHO1 and ANKRD31 and MEI1—form focal axis-bound complexes, called pre-DSB recombinosomes, which are thought to provide the molecular environment for SPO11-mediated DNA breakage on axes (12,15–20). Axis-associated DNA ends resulting from multiple DSBs along each chromosome promote homology search and the formation of synaptonemal complexes (SCs), which consist of parallelly aligned and juxtaposed pairs of homologue axes. SCs provide a permissive molecular context for efficient DSB repair and the generation of at least one crossover per homologue pair (1). Meiocytes control the numbers and the distribution of DSBs for effective homologue synapsis while preventing the formation of excessive DSBs that are redundant and/or defunct for promoting synapsis (1,9,12). Consistent with the notion that SPO11 auxiliary proteins are critical regulators of SPO11 activity, their expression and localization patterns conform to a predicted role in optimizing DSB patterns for homologue pairing and synapsis (15–18).

Pre-DSB recombinosomes form contemporaneously with the assembly of the axis shortly before the first DSBs appear at the start of meiosis. Despite a hypothesized essential role in enabling DSBs, pre-DSB recombinosomes do not colocalize with single-stranded DNA ends that result from DSBs (15). It is tempting to speculate that this puzzling lack of co-localization is caused by the disruption of pre-DSB recombinosomes in the vicinity of DSBs as part of a negative feedback mechanism that prevents excessive DSB formation. In mammals (21), Drosophila (22) and budding yeast (23–25), DSBs have been reported to inhibit SPO11 activity with the help of ATM (budding yeast Tel1), one of the three key DNA damage responsive phosphoinositide 3-kinase (PI3K)-related kinases (DDR PIKKs). ATM/Tel1 does not only limit total DSB numbers but seems particularly inhibitory towards the formation of adjacent DSBs, thereby promoting even distribution of DSBs for efficient homology search (24,26–28). DSB activity also correlates negatively with the accumulation of the pre-DSB recombinosome component Rec114 on axial sites in budding yeast (23). Given these observations, it is conceivable that ATM-mediated DSB suppression may involve a disruption of pre-DSB recombinosomes, manifesting as a lack of overlap between pre-DSB recombinosomes and cytological markers of DSBs on axes in mammals.

Whereas pre-DSB recombinosomes are distributed across all axes at the start of meiosis in the leptotene stage, they are restricted to unsynapsed axes as homologues pair and SCs starts to form in zygotene (15,16). This phenomenon may reflect an important negative feedback regulation of DSBs: DSBs promote SC formation, which, in turn, restricts SPO11 activity to unsynapsed sections of chromosome axes, where further DSBs are functional for homology search. The underlying mechanism likely involves a meiotic HORMA-domain protein, HORMAD1, which enhances both DSB formation and axial accumulation of pre-DSB recombinosomes (16,29). HORMAD1 localizes continuously along unsynapsed axes and recruits IHO1, thereby generating an axially-spread IHO1 platform, on which focal pre-DSB recombinosomes assemble (16). SC formation triggers the remodeling of HORMAD1 by TRIP13 AAA+ ATPase, leading to HORMAD1 depletion from synapsed axes (11). HORMAD1 depletion may underlie the inhibition of SPO11 activity in synapsed regions by diminishing axial recruitment of IHO1 and the dependent pre-DSB recombinosomes. Notably, in the absence of HORMAD1, sporadic pre-DSB recombinosomes and DSBs still form, and synapsis still promotes the depletion of the residual pre-DSB recombinosomes (16). Thus, the SC seems to inhibit pre-DSB recombinosomes by both HORMAD1-dependent and -independent mechanisms.

It appears that SC formation is not the only mechanism that disrupts pre-DSB recombinosomes to terminate SPO11 activity after the completion of homologue pairing. Whereas full synapsis of autosomes marks the onset of the pachytene stage in spermatocytes, heterologous sex chromosomes remain unsynapsed apart from their short homologous pseudoautosomal regions (PARs). Curiously, pre-DSB recombinosome components are depleted from unsynapsed axes shortly after the onset of pachytene by an unknown mechanism (15,17). Thus, pre-DSB recombinosomes are disrupted even on unsynapsed axes once DSBs no longer serve a purpose in promoting pairing and synapsis between homologues.

In summary, the spatiotemporal control of pre-DSB recombinosome components exhibit several features that support the restriction of SPO11 activity to regions of chromatin and stages of meiotic prophase where DSBs are productive for homology search. Yet, the mechanisms of pre-DSB recombinosome control are poorly understood. Given the central role of IHO1 in DSB formation and pre-DSB recombinosome assembly, we examined the characteristics and upstream factors of IHO1 regulation. Unexpectedly, we found three distinct negative feedbacks that inhibit IHO1 accumulation on chromosome axes in response to DSBs. Further, we found that not only ATM, but also the other two major DDR PIKKs, ATR and PRKDC, are involved in the DSB-induced disruption of the DSB machinery, albeit their roles are separable from ATM in these feedback mechanisms.

## MATERIALS AND METHODS

### Animal experiments

Genetically modified models were previously published: *Iho1* (16), *Iho1 flox* (16), *Spo11* (6), *Spo11 yf* (30), *Spo11 β-only* (31), *Mei4* (15), *Dmc1* (32), *Sycp1* (33), *Trip13* (34), *Spo11-Cre* (35), *Ngn3-Cre* (36), *Atr flox* (37), *Atm* (38), *Atm flox* (39), *Prkdc^scid^^/^^scid^* mice were obtained from the JacksonLabs. Littermate controls were used where possible. We applied a previously described strategy to generate ATR-deficient, ATR ATM double-deficient, and ATR ATM PRKDC triple-deficient mice using *Ngn3-Cre* transgene (40). Procedures pertaining to animal experiments were approved by the Governmental IACUC (Landesdirektion Sachsen) and overseen by the animal ethics committee of the Technische Universität Dresden. The licence numbers concerned with the present experiments with animals are DD24–5131/287/1, TV A 8/2017 and TVV 73/2017.

### Antibodies

Antibodies to IHO1, SYCP3, SYCP1, MEI4, H1t were produced and used as described in previous studies (16,17). Other antibodies used in this study were as follows: DMC1 (sc-22768, Santa Cruz, 1:100), RAD51 (sc-8349, Santa Cruz, 1:100), MLH1 (#3515, Cell Signaling, 1:50), RPA (#2208, Cell Signaling, 1:100), yH2AX (#05-636, Millipore, 1:2500).

### Nuclear surface spreads preparation and Immunofluorescence

Preparation and immunostaining of nuclear surface spreads of spermatocytes were performed similarly to what was described earlier with minor modifications (41). Briefly, testes were minced with forceps in PBS pH 7.4. The testis suspension was left standing for few minutes to allow sedimentation of large seminiferous tubule fragments. Supernatant was collected and centrifugated for 5 min at 1000 × g. Cell pellet was resuspended in PBS pH 7.4 and mixed with hypotonic extraction buffer (30 mM Tris–HCl pH 8.2, 50 mM sucrose pH 8.2, 17 mM sodium citrate pH 8.2, 5 mM EDTA pH 8.2, 0.5 mM DTT) in 1:1 ratio and incubated for 8 min at room temperature. After diluting the cell suspension five times with PBS pH 7.4, it was centrifuged for 5 min at 1000 × g. Cell pellet was resuspended in a 1:2 mixture of PBS and 100 mM sucrose solution. Cell suspensions were added to seven times higher volume droplets of filtered (0.2 μm) 1% paraformaldehyde (PFA), 0.15% Triton X-100, 1 mM sodium borate pH 9.2 solution on diagnostic slides and incubated for 1 h at room temperature. Nuclei were then dried for at least 1 h in a fume hood. Finally, the slides were washed in 0.4% Photo-Flo (Kodak) and were dried completely at room temperature.

For immunostaining of nuclear surface spread, slides were blocked with 2.5% (w/v) BSA in PBS for 30 min, then slides were incubated with primary antibodies diluted in blocking solution either for 3 h at room temperature or overnight at 4°C. Subsequently, slides were washed (3 × 10 min) with PBS and incubated with secondary antibodies at room temperature for 90 min. Finally, slides were washed (3 × 10 min) with PBS and mounted with Slowfade gold antifade mounting media with or without DAPI (Invitrogen).

### Testis cryosections preparation and immunofluorescence

Testes were collected in PBS pH 7.4 and fixed for 40 min at room temperature in 4% formaldehyde, 100 mM sodium phosphate pH 7.4, 0.1% Triton-X. After fixation, testes were washed three times in PBS and incubated in 30% sucrose, 0.02% sodium azide overnight at 4°C. Afterwards, testes were frozen in OCT on dry ice and stored at –80°C until sectioned. After cryo-sectioning, 7 μm sections were dried onto glass slides, incubated in cold methanol for 10 min at –20°C and subsequently in cold acetone for 1 min at –20°C. Sections were finally washed in PBS and blocked with PBS pH 7.4, 10% goat serum, 0.05% tween and 0.05% Triton-X. Slides were incubated with primary antibodies diluted in blocking solution overnight at 4°C. Subsequently, slides were washed (3 × 10 min) with PBS 0.01% Tween and incubated with secondary antibodies at room temperature for 90 min. Finally, slides were washed (3 × 10 min) with 0.01% Tween and mounted with Slowfade gold antifade mounting media with DAPI (Invitrogen).

### Staging of meiotic prophase

Nuclear spreads were staged based on axis development, which was assessed through detection of SYCP3 on meiocyte spreads. Briefly, punctate SYCP3 staining corresponds to preleptotene, short stretches of axis correspond to leptotene (most axis stretches shorter than 5× the widths of axes), relatively long and incomplete stretches of axes corresponds to early-zygotene and complete axes corresponds to late-zygotene and pachytene stages. Late zygotene and early pachytene could be distinguished unambiguously only in wild type where SC formation is efficient. In wild type, the completion of SC marks the start of pachytene. To identify sub-stages in wild-type pachytene spermatocytes (Supplementary Figures S5 and S6), we used the combination of SYCP3 and H1T (42) staining. In zygotene-to-pachythene transition all autosomes appear synapsed, but the very ends of autosome axis pairs appear thicker, indicating that synaptonemal complex formation just finished; no H1T staining is observed. In early-pachytene the thickness of synapsed axis-pairs is largely uniform along their entire length, the synapsed PAR region is long (length is >5× the widths) or short (length is <5× but >3× longer than the widths) and there is no or there is only very weak histone H1T signal. The above two sub-stages can be distinguished by IHO1 staining as IHO1 accumulates on the PAR region during zygotene-to-pachythene transition while disappears completely in early pachytene as shown earlier (16). In late-pachytene, the synapsed axes of autosomes are clearly thicker at their ends, and histone H1T signal intensity is intermediate to strong. Diplotene is characterized by desynapsing axes, thickened axes at chromosomal ends and high histone H1T levels.

### Staging of mouse seminiferous tubule cross sections

To stage the epithelial cycle of mouse seminiferous tubules, we used criteria that were described earlier (43,44). Specifically stages V–VI are characterized by a basal cell layer containing spermatogonia B (oval shaped nuclei with multiple round-shaped DAPI-bright heterochromatic regions) and a second cell layer consisting of mid pachytene cells, which express intermediate levels of histone H1t.

### Testis organ culture and irradiation

Testis organ culture protocol was carried out as described earlier (45) with minor modifications. Testes were dissected and tunica albuginea was removed. Freshly isolated tubules were irradiated (Gammacell 3000 Elan) and cultured at gas/liquid interphase on agarose gel blocks (1,5%; w/v; thickness about 7 mm). Gel pieces were preincubated in culture medium for 24 h to saturate agarose with medium before irradiated samples were placed on them. After the biological samples were placed on the agarose slices, the medium level was adjusted not to cover the seminiferous tubules in the culture wells. We used α-MEM (Life Technologies), 10% (v/v) KSR (Life Technologies) and gentamycin (Sigma-Aldrich) at a final concentration of 5 μg/ml as culture medium. Tubules were incubated at 34°C in humidified atmosphere containing 5% CO_2_.

### IHO1 and HORMAD1 intensity measurements on synapsed and unsynapsed axes

ImageJ was used to process images for immunofluorescence signal measurements (46,47). Partially synapsed chromosome axes were identified. Synapsed and unsynapsed regions were marked separately using ‘Freehand selection’ tool. Two empty areas were also selected nearby in order to calculate background. Intensity values were obtained manually using ‘Measure’ command.

### IHO1 and DMC1/RAD51 intensity for correlation

ImageJ was used to process images for immunofluorescence signal measurements and R was used to analyse acquired data (46–48). In the images of most cells, we selected 5–12 unsynapsed chromosome axes where both IHO1 and DMC1 or RAD51 were present. Selections were made manually. The ‘Freehand line’ tool was used with 5 pixels width in order to cover the entire axis thickness. Only chromosome axes not overlapping with others were selected. Care was taken to ensure that the selected line overlapped with all axis associated-RAD51/DMC1 foci even if they were offset from the center of axes. All selections were added to ROI Manager (‘Region of Interest, ROI’). A custom macro using getProfile function was prepared to obtain intensities of different channels from the same selection. getProfile function averages intensities over the line width (see macro in Supplementary Methods). Background was measured in each cell in 2–3 nuclear region that did not overlap with axes. Background subtracted IHO1 and corresponding DMC1 or RAD51 signals are provided as Supplementary Data file 1 and 2.

IHO1, RAD51 and DMC1 signals were scaled and centered in each axis fragment by the deduction of the mean and division by the standard deviation. Auto-correlation and cross-correlation functions were computed using the R function ccf. For each experiment and for each fragment we computed:

$ < {s_i}{s_j}{ >_f}$ and $ < {s_i}{r_j}{ >_f}$, where ${s_i}$ is the value of the scaled signal for a given protein (e.g. IHO1), ${s_j}$ is the value of the scaled intensity of the same protein at position j in pixels in case of auto-correlation, $c( x )$, and ${r_j}$ is the scaled intensity of a different protein species in case of cross-correlation,$\ g( x )$. $ < \ldots { >_f}$ denotes the average over fragments. After computing the expected values of auto-correlations and cross-correlations for each fragment, the final values are computed by averaging over all the fragments from all cells of a given genotype,

$$\begin{equation*}c\ \left( x \right) = \mathop \sum \limits_{f = 1}^{\# fragments} < {s_i}{s_j}{ >_f}/\# fragments\ ,\end{equation*}$$

$$\begin{equation*}g\left( x \right),\ = \mathop \sum \limits_{f = 1}^{\# fragments} < {s_i}{r_j}{ >_f}/\# fragments\ .\end{equation*}$$

*P*-values are computed using a *t*-test (*t*-test function in R) for each given distance where each sample is given by the value of ${s_i}{s_j}$ or ${s_i}{r_j}$. Tested is the null hypothesis that scaled protein levels are uncorrelated, $c( x ),\ g\ ( x ) = \ 0$.

### Statistics and reproducibility

Graphs were prepared using GraphPad Prism 5 and all types of the statistical tests and *P*-values are indicated in the corresponding figure legends. Experiment quantifications and conclusions in the manuscript are based on results that were reproduced in at least two independent experiments and at least two mice of each genotype. All comparisons were made between datasets obtained from animals that were either littermates or age matched.

### Biological materials availability

Transgenic mouse strains, analysis scripts and pipelines used in this study are available from the authors upon request.

## RESULTS

### IHO1 is locally removed in the vicinity of DNA double-strand breaks

**Figure 1.**
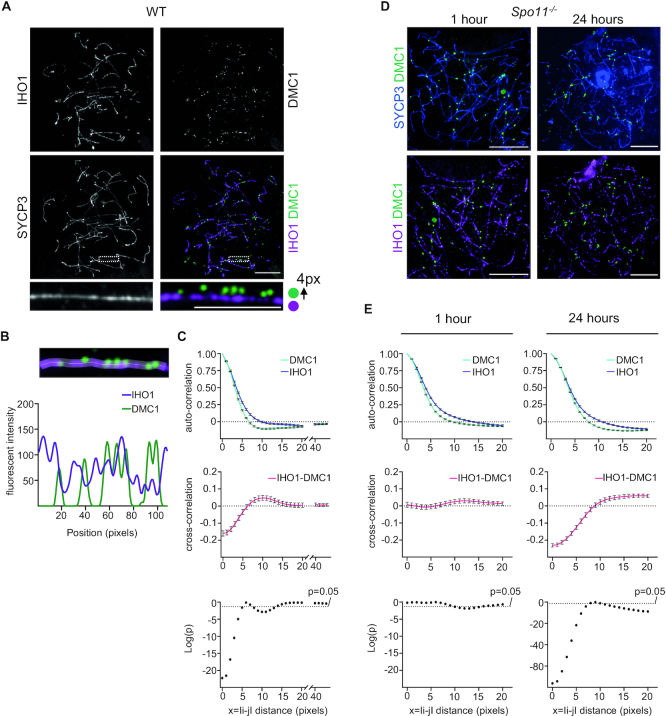
Depletion of axial IHO1 in the vicinity of DSBs. (**A, D**) IHO1 and markers of the chromosome axis (SYCP3) and unrepaired DSBs (DMC1) were detected in nuclear surface spread spermatocytes of adult wild-type (**A**) or *Spo11^−^^/^^−^* (**D**) mice by immunofluorescence. The *Spo11^−^^/^^−^* spermatocytes **(D)** were exposed to 4Gy γ-irradiation and, subsequently, cultured for 1 or 24 h *in vitro* before nuclear spread preparation. In (**A**), enlarged insets (bottom) show a section of IHO1-rich unsynapsed chromosome axis. DMC1 signal was shifted 4 pixels up in the inset of the overlay image to help comparison between the distribution of DMC1 and IHO1 signals. Bars, 10 μm in main and 5 μm in enlarged inset. (**B**) Quantification of axis-associated IHO1 and DMC1 immunofluorescence signals along the axis section that is shown in the enlarged insets in (**A**). Signal intensity is shown in arbitrary units. Position is indicated in pixels from left to right along the axis. (**C, E**) Correlation between IHO1 and DMC1 signals along unsynapsed chromosome axes of untreated wild type (**C**) or γ-irradiated *Spo11^−^^/^^−^* (**E**) spermatocytes. Measurements of IHO1 and DMC1 signals took place in *Spo11^−^^/^^−^* spermatocytes that were cultured for 1 or 24 h after 4 Gray γ-irradiation. (**C, E**) Correlations were calculated between signals in all positions along the length of axis sections. Top and middle graphs show auto-correlations of IHO1 (blue) and DMC1 (green) signals (top graphs) or cross-correlation between IHO1 and DMC1 signals (middle graphs, red). Averages of correlations are shown for every axial distance between 0 and 20 pixels in (**C**) and (**E**), and between 40 and 43 pixels in (**C**), *x* = I*i* – *j*I represents distances between *i* and *j* positions along axis sections, averages of measurements from multiple axis segments of spermatocytes of two mice are shown (see supplementary data file 1 for raw values), error bars indicate standard error of the mean. Bottom graphs show significance of cross-correlation on base 10 logarithmic scale. Graphs are based on the analyses of *n* = 26 cells and *n* = 217 axes in (**C**); *n* = 29 cells and *n* = 382 axes for the 1h sample and *n* = 38 cells and *n* = 488 axes for the 24 h sample in (**E**).

According to current models of DSB formation, pre-DSB recombinosome-rich sections of axis accumulate DSBs. In turn, DSBs inhibit further DSB formation in their vicinity possibly by promoting a local disruption of pre-DSB recombinosomes (15). IHO1 accumulates on unsynapsed axis and forms a discontinuous axial platform, on which focal MEI4-REC114-rich pre-DSB recombinosomes assemble (16). Given a hypothesized inhibition of the DSB machinery by DSBs, a discontinuous IHO1 distribution on axis may reflect not only uneven deposition of IHO1 but also a depletion of IHO1 in the vicinity of DSBs. To test this hypothesis, we compared the distribution of IHO1 and DSB markers DMC1 or RAD51 along unsynapsed sections of axes (Figure 1A and Supplementary Figure S1A, IHO1 staining was used to identify unsynapsed chromosome axis regions). This comparison revealed an apparent relationship between IHO1 and DMC1 or RAD51. Although most DMC1 and RAD51 foci were in the vicinity of elevated IHO1 signal, the peaks of DMC1/RAD51 signals were mostly associated with locally reduced IHO1 signal (Figure 1B and Supplementary Figure S1B).

It is not straightforward to quantify and interpret the relationship between IHO1 and DMC1/RAD51 levels along axes. IHO1-rich axis sections do not inevitably accumulate DSBs because the axial IHO1 platform does not always associate with pre DSB recombinosomes (16). Further, according to our hypothesis, IHO1-negative axis sections may arise both due to a lack of IHO1 deposition and due to depletion triggered by DSBs. Therefore, we do not expect a strong correlation between levels of IHO1 and DSB markers in relation to the position along unsynapsed axes. To permit statistical comparisons of IHO1/DMC1/RAD51 signal profiles from multiple unsynapsed axes of several cells, we first scaled signals by subtracting the mean and dividing by the standard deviation of signal values for each analysed axis section. Thereafter, we calculated both auto- and cross-correlation between scaled IHO1/DMC1/RAD51 immunofluorescence signals for all possible distances between two positions along the length of each examined axis, $c( x )$ and $g( x )$, respectively, where $x\ = \ | {i - j} |$ is the distance between pixel positions $i$ and $j$ along unsynapsed chromosome axes, and 1 pixel corresponds to 65 nm distance on axes. The values of the auto-correlation function quantify the spatial distributions of IHO1, RAD51 and DMC1 along the axis, while the cross-correlation function quantifies the spatial relationship between the different signals. We noticed that a positive auto-correlation of IHO1, RAD51 and DMC1 between nearby positions gradually turned into a negative auto-correlation beyond 5-pixel distances (*x* > 5) (Figure 1C and Supplementary Figure S1C). This negative auto-correlation indicates that, DMC1/RAD51/IHO1 signal peaks are often accompanied by valleys as one moves along the axis beyond 5 pixel distances (>325 nm), The negative auto-correlation of DMC1/RAD51 signal was particularly obvious, which is consistent with, the observation that DMC1/RAD51 peaks mostly span 8–12 pixels. As the distances increased well beyond 20 pixels, the auto-correlation approached 0 (Figure 1C and Supplementary Figure S1C). In contrast to the auto-correlation, we observed a highly significant negative cross-correlation between IHO1 and DMC1, *g*(*x*) = –0.16054 and *P* = 5.67E–23, or RAD51, *g*(*x*) = –0.16592 and *P* = 8.76E–30, in the same pixel positions (*x* = 0). The negative cross-correlation was gradually lost with increasing distance and, curiously, turned into a weak but significant positive cross-correlation beyond 5-pixel distances (x>5) (Figure 1C and Supplementary Figure S1C). These positive IHO1-DMC1 and IHO1-RAD51 cross-correlations at a distance may result from the combined effects of negative IHO1-DMC1 and IHO1-RAD51 cross-correlations in the same positions (*x* = 0) and negative-auto-correlations of IHO1, RAD51 and DMC1 at a distance *x* > 5. With further increase of the distance (*x* > 15) the IHO1-DMC1 and IHO1-RAD51 cross-correlations approached 0.

These data suggest that RAD51 and DMC1 peaks occur preferentially within IHO1 valleys and that there is a 5–10-pixel offset (0.3–0.65 μm) between peaks of IHO1 signals relative to DMC1 or RAD51 peaks. Thus, the observed patterns conform with the hypothesis that DSBs form in sections of axis where pre-recombinosome components accumulate, but after their formation, DSBs may promote the local depletion of pre-DSB recombinosome components from the axis. The observations are also formally consistent with additional non-exclusive possibilities. DSBs may form next to, and not within, IHO1-rich axis sections, or DSBs may form in IHO1-rich regions, but resultant resected DNA ends associate preferentially with nearby IHO1-poor axis regions.

To test these alternatives, we aimed to analyse the relationship between axial distributions of DSB foci and IHO1 in an experimental system where DSB formation is inducible and does not depend on IHO1. These criteria are fulfilled by γ-irradiation induced DSBs. Whereas there may be differences in the processing of SPO11-induced and γ-irradiation-induced DSBs in meiosis, both γ-irradiation- and SPO11-induced DSBs produce DNA ends that associate with chromosome axes and recruit RAD51 and DMC1 (49). These properties of γ-irradiation-induced DSBs permitted us to assay the relationship between the position of induced DSBs and IHO1 on axes. We introduced exogenous DSBs in *Spo11^−^^/^^−^* spermatocytes by γ-irradiation and assayed DSBs 1 and 24 h later. In line with an earlier study (49) that detected single-stranded DNA ends on axes of *Spo11^−^^/^^−^* spermatocytes after DSB induction by γ-irradiation, we observed that at both time points DMC1 foci were primarily on axes (Figure 1D). γ-irradiation is expected to induce most DSBs off axes, as the axis contains only a small fraction of the genome in each cell. Thus, the preferential axis association of DMC1 foci indicates that new DNA ends that result from irradiation are recruited to axes in spermatocytes. Hence, we would expect a loss of IHO1 in the vicinity of DMC1 foci as time passes if DSBs and/or initial DSB repair steps induce local depletion of IHO1 during meiosis. Interestingly, 1 h post-irradiation, there was no significant cross-correlation between IHO1 and DMC1 between the same (*i* = *j*) or distinct positions (*i* ≠ *j*). This indicates that irradiation-induced DNA ends do not associate preferentially with IHO1-poor regions (Figure 1E). Importantly, 24 h after the irradiation, the patterns of IHO1-DMC1 cross-correlation in *Spo11^−^^/^^−^* spermatocytes resembled the patterns observed in unirradiated wild-type spermatocytes. Thus, there was a highly significant negative cross-correlation between IHO1 and DMC1 in the same positions (*i* = *j*, *g*(0) = –0.23051, *P* = 6.07E–77). This observation supports the hypothesis that DSBs and/or single-stranded DNA-ends resulting from DSBs promote local depletion of IHO1 from axis, which may contribute to a negative feedback mechanism that prevents formation of DSBs in each other′s proximity.

### ATR activity is required for local IHO1 depletion upon DSB formation

Negative feedback control of DSBs is thought to involve the ATM kinase. Accordingly, ATM suppresses DSB numbers by ∼10-fold in mice (21). Hence, we tested if ATM was involved in the observed IHO1 depletion near DSBs. Due to excessive numbers of DSBs, *Atm^−^^/^^−^* spermatocytes have pleiotropic phenotypes including chromosome axis fragmentation and an abnormal chromatin-wide hyperaccumulation of a marker of unrepaired DSBs, phospho-histone H2AX (Ser139), also called γH2AX (25,50). These pleiotropic effects are absent or strongly reduced in *Atm^−^^/^^−^ Spo11^+/^^−^* background where DSB numbers are reduced to about half relative to *Atm^−^^/^^−^* (21). Hence, we compared *Atm^−^^/^^−^ Spo11^+/^^−^* and *Spo11^+/^^−^* spermatocytes to assess the effect of ATM-deficiency on cross-correlation between IHO1 and DMC1/RAD51.

**Figure 2.**
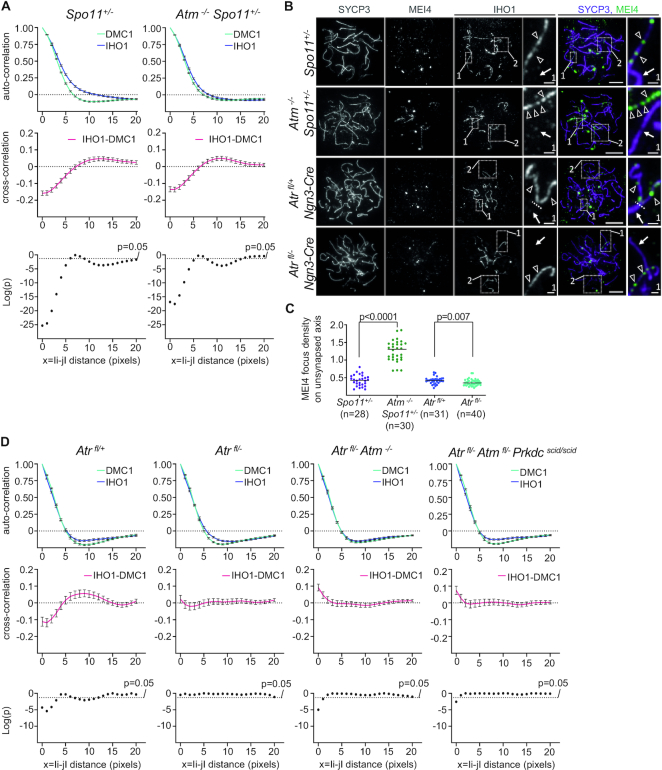
ATM- and ATR-deficiencies differentially affect pre-DSB recombinosome and IHO1 distributions on axes. (**A, D**) Correlation between IHO1 and DMC1 signals along unsynapsed chromosome axes in spermatocytes of indicated genotypes. Correlations were calculated between signals in all positions along the length of axis sections. Top and middle graphs show auto-correlations of IHO1 (blue) and DMC1 (green) signals (top graphs) or cross-correlation between IHO1 and DMC1 signals (middle graphs, red). Averages of correlations are shown for every axial distance between 0 and 20 pixels, *x* = I*i* – *j*I represents distances between *i* and *j* positions along axis sections, error bars indicate standard error of mean. Bottom graphs show significance of cross-correlation on base 10 logarithmic scale. Graphs are based on the analyses of *n* = 35 cells and *n* = 371 axes for *Spo11^+/^^−^* (**A**), *n* = 34 cells and *n* = 348 axes for *Atm^−^^/^^−^Spo11^+/^^−^* (**A**), *n* = 26 cells and *n* = 294 axes for *Atr^fl^^/+^* (**D**), *n* = 48 cells and *n* = 354 axes for *Atr^fl^^/^^−^* (**D**), *n* = 49 cells and *n* = 457 axes for *Atr^fl^^/^^−^Atm^−^^/^^−^* (**D**), *n* = 38 cells and *n* = 352 axes for *Atr^fl^^/^^−^Atm^fl^^/^^−^Prkdc^scid^^/^^scid^* (**D**). (**B**) IHO1 and markers of the chromosome axis (SYCP3) and the pre-DSB recombinosome (MEI4) were detected in spread spermatocytes of indicated genotypes. Enlargements of selected regions 1 and 2 are shown in (**B**) and Supplementary Figure S2B, respectively, for each genotype. Enlarged insets (region 1) show synapsed and unsynapsed chromosome regions as identified by the absence or presence of IHO1, respectively. Arrows mark synapsed axes. Triangles mark MEI4 foci on unsynapsed axes. Dotted lines indicate the border between synapsed and unsynapsed regions of a partially synapsed chromosome in the insets from *Atr^fl^^/+^ Ngn3-Cre* spermatocyte. Bars, 10 μm in main and 1 μm in enlarged insets. (**C**) Quantification of MEI4 focus densities (focus/μm) on unsynapsed axes of late-zygotene or zygotene-pachytene like spermatocytes of indicated genotypes. Numbers of analyzed cells (*n*) and medians (bars) are indicated. Pool of two experiments and results of Mann–Whitney *U* test are shown.

Interestingly, we found a significant negative cross-correlation between IHO1 and DMC1/RAD51 in the same positions (*i* = *j*, IHO1-DMC1 *g*(0) = –0.13533 and *P* = 1.38E–17, IHO1-RAD51 *g*(0) = –0.11507 and *P* = 1.91E-16) in *Atm^−^^/^^−^ Spo11^+/^^−^* spermatocytes resembling *Spo11^+/^^−^* or wild-type spermatocytes (Figure 2A and Supplementary Figure S2A). Thus, depletion of IHO1 in the vicinity of DSBs is unlikely to play a major role in ATM-mediated DSB control. As a corollary, IHO1 depletion likely represents a feedback mechanism of the DSB machinery that is largely independent of ATM.

Unlike IHO1 localization, MEI4 localization on axes was strongly affected by ATM-deficiency (Figure 2B). We observed 3-fold elevation of MEI4 density along unsynapsed axes of late zygotene spermatocytes in *Atm^−^^/^^−^ Spo11^+/^^−^* as compared to *Spo11^+/^^−^* or wild-type mice (Figure 2C, unsynapsed axes were identified by the presence of IHO1). Hence, ATM-mediated DSB control may involve disruption of MEI4-REC114-IHO1 complexes on axis.

Given that ATM deficiency had little effect on the distribution of IHO1 in the vicinity of DSBs, we tested if other DNA damage-regulated PIKKs, ATR and PRKDC, had an effect. To test if ATR played a role, we conditionally disrupted *Atr* in the spermatogenic lineage using a previously reported mouse line (*Atr^−^^/^^fl^ Ngn3-Cre*, (40)). ATR-deficient spermatocytes efficiently load IHO1 on axes (Figure 2B) and efficiently form DSBs, but are thought to inefficiently load RAD51 and DMC1 on single-stranded DNA resulting from DSBs (40,51). Nevertheless, DMC1 and RAD51 foci still form in significant numbers (more than 50% of wild type) in ATR-deficient meiocytes (40,51), which permits comparisons between the spatial distributions of DMC1, RAD51 and IHO1 on chromosome axes. Whereas ATR deficiency did not lead to increased MEI4 density along unsynapsed axes (Figure 2B, C and Supplementary Figure S2B), and it did not cause major alterations in the auto-correlation of IHO1, DMC1 or RAD51 signals, it did cause a complete loss of IHO1 cross-correlation with DMC1 and RAD51 (*i* = *j*, IHO1-DMC1 *g*(0) = 0.022522 and *P* = 0.338988, IHO1-RAD51 *g*(0) = 0.012415 and *P* = 0.260504), suggesting an important role for ATR in IHO1 depletion in the vicinity of DSBs (Figure 2D and Supplementary Figure S2C). Further, we found that ATM deletion in *Atr^−^^/^^fl^ Ngn3-Cre* spermatocytes caused a weak but significant positive cross-correlation between IHO1 and DMC1/RAD51 in the same positions (*i* = *j*, IHO1-DMC1 *g*(0) = 0.09151 and *P* = 1.01E–05, IHO1-RAD51 *g*(0) = 0.07206 and *P* = 9.39E-11) along the axis. The hypomorphic *scid* mutation of *Prkdc* (52) did not result in further positive cross-correlation in an ATM-ATR-double deficient background, which is consistent with the idea that PRKDC plays only minor roles in meiotic recombination between leptotene and mid pachytene (35,38). These results suggest that DSBs promote depletion of IHO1 in their vicinity primarily by an ATR-dependent mechanism, while ATM plays a minor role, particularly in the absence of ATR. Hence, not only ATM, but also ATR seem to mediate feedback control on the DSB machinery in mammals.

### TRIP13 is required for IHO1 depletion in response to synapsis but not in response to DSB formation or pachytene onset

**Figure 3.**
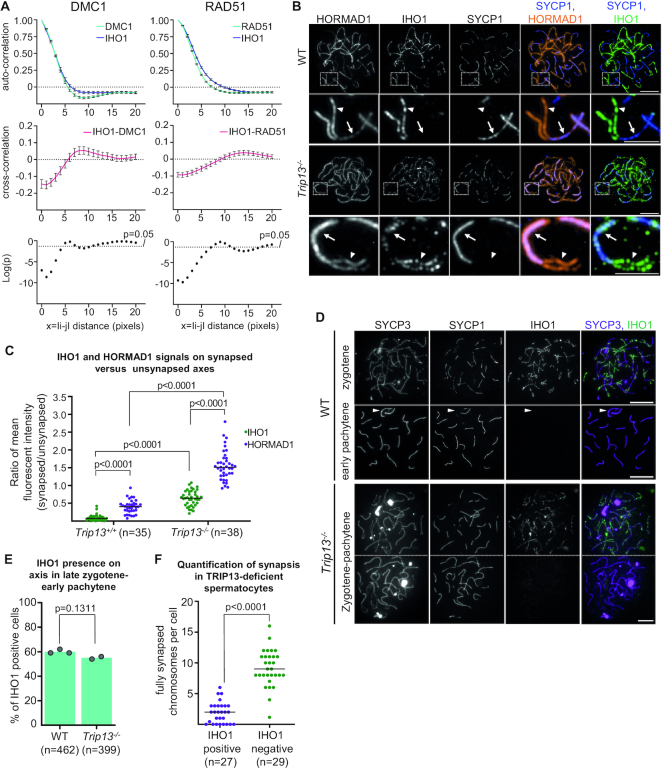
TRIP13 deficiency impairs IHO1 depletion from synapsed axes. (**A**) Correlation between IHO1 and DMC1 or RAD51 signals along unsynapsed chromosome axes in TRIP13-deficient spermatocytes. Correlations were calculated between signals in all positions along the length of axis sections. Top and middle graphs show auto-correlations of IHO1 (blue) and DMC1 or RAD51 (green) signals (top graphs) or cross-correlation between IHO1 and DMC1 or RAD51 signals (middle graphs, red). Averages of correlations are shown for every axial distance between 0 and 20 pixels, *x* = I*i* – *j*I represents distances between *i* and *j* positions along axis sections, error bars indicate standard error of the mean. Bottom graphs show significance of cross-correlation on base 10 logarithmic scale. Graphs are based on the analyses of *n* = 16 cells and *n* = 145 axes for DMC1-IHO1 correlation, *n* = 18 cells and *n* = 273 axes for RAD51-IHO1 correlation. (**B, D**) IHO1, synapsis (SYCP1) and either HORMAD1 (**B**) or chromosome axes (**D**, SYCP3) were detected in late zygotene or late zygotene-like spermatocytes of adult wild-type or *Trip13^−/−^* mice, respectively. (**B**) Enlarged insets show partially synapsed chromosomes where synapsed and unsynapsed sections are marked by arrows and arrowheads, respectively. (**D**) Spermatocytes are shown in the indicated prophase stages. Arrowhead indicates the partially synapsed X and Y sex chromosomes in the wild-type early pachytene spermatocyte. In the *Trip13^−^^/^^−^* panel, two adjacent cells are separated by a dotted line; the upper cell has only one fully synapsed chromosome, the lower cell has seven fully synapsed chromosomes. (**B, D**) Bars, 10 μm in main panels and 5 μm in enlarged insets. (**C**) Graph shows ratios of mean IHO1 (green) or HORMAD1 (purple) signal intensities on unsynapsed axes vs. synapsed axes in late zygotene (wild type) or late zygotene-like (*Trip13^−^^/^^−^*) spermatocytes. Numbers of analyzed chromosomes (n) per category is indicated. Results of Mann–Whitney *U* test are shown. (**E**) Quantification of IHO1 presence on axes in late zygotene-early pachytene wild-type (WT) and *Trip13^−/−^* spermatocytes, which were identified based on their fully developed axes and an absence of the post-early pachytene marker histone H1t. Graph shows the proportion of IHO1 positive spermatocytes. Block bars show averages of two experiments, total numbers of counted cells are indicated. A likelihood ratio test indicated no significant difference (*P* = 0.1311). **(F)** Quantification of fully synapsed chromosomes in two non-overlapping groups of late zygotene-early pachytene *Trip13^−/−^* spermatocytes where IHO1 was either present or absent on unsynaped axes. Numbers of analyzed cells (*n*) and medians (bars) are indicated. Median numbers of fully synapsed chromosomes were two and nine in IHO1 positive and negative cells, respectively. Mann Whitney U test indicated a significant difference (*P* < 0.0001).

TRIP13 promotes depletion of HORMAD1 from synapsed axes (11,34), possibly by directly remodeling HORMAD1 (53). Previously, we hypothesized that HORMAD1 depletion from synapsed chromosomes may restrict DSB formation to unsynapsed axes (11,29), where continued DSB activity may be needed to complete homologue pairing. Given that IHO1 depends on HORMAD1 for efficient axis association (16), we explored if TRIP13 also regulated IHO1 distribution on chromosome axes. IHO1-DMC1 and IHO1-RAD51 cross-correlation plots indicated significant IHO1 depletion in the vicinity of DSBs on unsynapsed axes in spermatocytes of a TRIP13-deficient mouse line (originally described as *Trip13^sev/^^sev^* in (34), hereafter *Trip13^−^^/^^−^*), resembling wild type (Figure 3A). Thus, TRIP13 does not play a major role in DSB-induced depletion of IHO1 from unsynapsed axes.

In contrast, TRIP13 had an important role in the depletion of IHO1 from synapsed axes. Whereas IHO1 was always absent from synapsed axes in wild type, IHO1 persisted on synapsed axes in all *Trip13^−^^/^^−^* spermatocytes where IHO1 was detectable on chromosomes (Figure 3B, Supplementary Figure S3A, S3B, synapsed axes were identified by the presence of SYCP1). This observation suggests that a failure to remodel HORMAD1 by TRIP13 leads to abnormal persistence of both HORMAD1 (53) and IHO1 on synapsed axes. Alternatively, TRIP13 may promote IHO1 depletion by both HORMAD1 depletion and a HORMAD1-independent mechanism. Regardless, these observations suggest that TRIP13-dependent loss of IHO1 from synapsed axes contributes to a negative feedback mechanism that shuts down DSB formation in regions of the genome where homologues successfully synapsed. Whereas HORMAD1 amounts were higher, IHO1 amounts were moderately lower on synapsed axis pairs than lone unsynapsed axes in *Trip13^−^^/^^−^* spermatocytes (Figure 3B, C, Supplementary Figure S3A, S3B). Further, whereas MEI4-containing recombinosomes appeared less efficiently depleted from synapsed regions in *Trip13^−^^/^^−^* spermatocytes as compared to wild type, the density of MEI4 foci was still 3-fold lower in synapsed than unsynapsed regions in *Trip13^−^^/^^−^* spermatocytes (Supplementary Figure S3C, D). Thus, synapsis seems to downregulate the DSB machinery by not only a TRIP13-dependent but also a TRIP13-independent mechanism.

Although IHO1 was not restricted to unsynapsed axes in the absence of TRIP13, IHO1 was often depleted from all synapsed and unsynapsed axes in *Trip13^−^^/^^−^* spermatocytes that had fully developed axes (Figure 3D; *Trip13^−^^/^^−^* cell in the bottom). We wondered if IHO1 depletion from all axes of *Trip13^−^^/^^−^* spermatocytes was analogous to the disappearance of IHO1 from all chromosome axes, including axes of unsynapsed XY chromosomes, upon pachytene entry in wild type (16). Fully developed axes are indicative of late zygotene and early pachytene stages in *Trip13^−^^/^^−^* spermatocytes. This is because axis formation is completed only after mid zygotene, and *Trip13^−^^/^^−^* spermatocytes are eliminated at the onset of mid pachytene due to defects in recombination and synapsis (34,36). Synaptonemal complex and axis morphology do not unambiguously distinguish late zygotene and early pachytene stages in synapsis-defective mutants, including *Trip13^−^^/^^−^*, hence we will refer to these stages as late zygotene-early pachytene hereafter. Interestingly, similar fractions of late zygotene-early pachytene spermatocytes lack IHO1 from all axes in wild-type (40%) and *Trip13^−^^/^^−^* (45%) mice (Figure 3E). Furthermore, IHO1-positive *Trip13^−^^/^^−^* spermatocytes had less synapsed chromosomes (Figure 3D, top *Trip13^−^^/^^−^* cell, quantification in Figure 3F) than IHO1-negative *Trip13^−^^/^^−^* spermatocytes (Figure 3D, *Trip13^−^^/^^−^* cell in the bottom, quantification in Figure 3F) indicating that IHO1 depletion occurs in the most advanced prophase stages in *Trip13^−^^/^^−^* spermatocytes. Hence, while we cannot unambiguously tell late zygotene and early pachytene stages apart in *Trip13^−^^/^^−^* spermatocytes, our observations suggest that IHO1 is depleted from *Trip13^−^^/^^−^* spermatocytes in an early pachytene-equivalent stage.

Thus, TRIP13 appears to be dispensable for IHO1 depletion from all axes in early pachytene. Furthermore, whereas SC and IHO1 dynamics in wild type suggest that IHO1 depletion from all axes is linked to the completion of autosomal synapsis at pachytene onset, the phenotype of *Trip13^−^^/^^−^* spermatocytes argues against this interpretation. Asynaptic *Trip13^−^^/^^−^* spermatocytes deplete IHO1 from synapsed and unsynapsed axes alike in an early pachytene-like stage, which indicates that synapsis completion is not a prerequisite for all-axis IHO1 depletion.

### All-axis depletion of IHO1 in pachytene requires DSB formation but not synapsis

**Figure 4.**
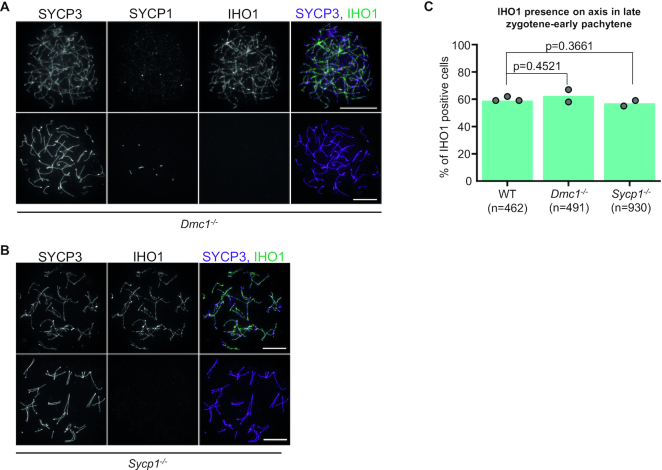
IHO1 depletion from all axes occurs in *Dmc1^−^^/^^−^* and *Sycp1^−^^/^^−^* spermatocytes in late zygotene-early pachytene. (**A, B**) IHO1 was detected in combinations with chromosome axes (SYCP3, both **A** and **B**) and synapsis (SYCP1 in **A**) in spread spermatocytes of adult (**A**) *Dmc1^−/−^* and (**B**) *Sycp1^−/−^* mice. Images show examples of IHO1 positive (upper panel) and negative (lower panel) spermatocytes in late zygotene-early pachytene stage. Bars, 10 μm. (**C**) Quantification of IHO1 presence on axes in late zygotene-early pachytene wild-type (WT), *Dmc1^−^^/^^−^* and *Sycp1^−/−^* spermatocytes. Late zygotene-early pachytene spermatocytes were identified based on their fully developed axes and an absence of the post-early pachytene marker histone H1t. Graph shows the proportion of IHO1 positive spermatocytes. Block bars show averages of two experiments, total numbers of counted cells are indicated. Wild-type samples are the same as in Figure 3D. A likelihood ratio test indicated no significant difference between wild-type and *Dmc1^−^^/^^−^* (*P* = 0.4521) or *Sycp1^−/−^* (*P* = 0.3661) spermatocytes.

We further tested if early steps in recombination or completion of synapsis are important for all-axis depletion of IHO1 in early pachytene. Hence, we examined various meiotic mutants where synapsis formation was defective due to impaired DSB formation (*Spo11^−^^/^^−^* mice described by (6)), impaired DSB repair (*Dmc1^−^^/^^−^* mice described by (32)) or an absence of the SC transverse filament protein SYCP1 (mice described in (33)) (Figures 4 and5). In all of these mutants, defects in SC and recombination trigger spermatocyte elimination in a mid pachytene-equivalent stage (25,36–37,37,54), and fully formed axes mark late zygotene-early pachytene stages. We found that similar fractions of late zygotene-early pachytene spermatocytes depleted IHO1 from all chromosome axes in wild type (40%), DSB repair-defective *Dmc1^−^^/^^−^* (38%) and SC-defective *Sycp1^−^^/^^−^* (43%) mice (Figure 4C). This suggests that neither asynapsis nor DSB repair defects prevent depletion of IHO1 from chromosome axes in an early pachytene-equivalent stage.

**Figure 5.**
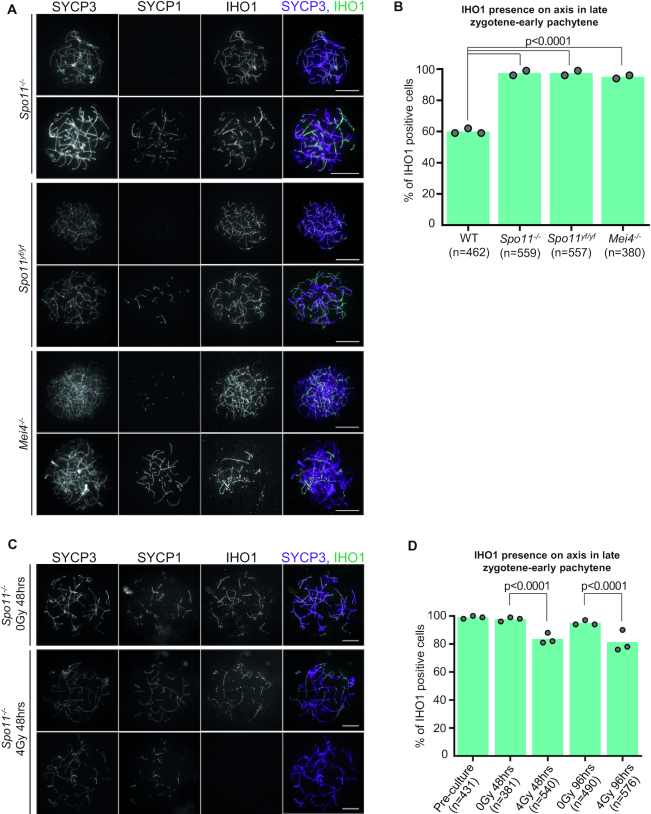
All-axis depletion of IHO1 depends on DSBs. (**A, C**) IHO1 and markers of the chromosome axis (SYCP3) and synapsis (SYCP1) were detected by immunofluorescence in nuclear surface spread spermatocytes of indicated genotypes. Spermatocytes are shown in zygotene-early pachytene stage. Bars, 10 μm. (**C**) Nuclear spreads of *Spo11^−^^/^^−^* spermatocytes that were cultured for 48 h *in vitro* either following an exposure to 4 Gray γ-irradiation or without γ-irradiation. (**B, D**) Quantification of IHO1 presence on axes in late zygotene-early pachytene spermatocytes of indicated genotypes (**B**) or in late zygotene-early pachytene Spo11^−/−^ spermatocytes that were cultured for 48 or 96 h after 0 or 4 Gray γ-irradiation (**D**). Graphs show the proportion of IHO1 positive spermatocytes. Block bars show averages of two (**B**) or three (**D**) experiments, total numbers of counted cells are indicated. A likelihood ratio test calculated *P* < 2.2e–16 for differences between the indicated genotypes and wild type (wild type is the same as in 3D and 4C) in (**B**), and *P* values for differences between irradiated and unirradiated *Spo11^−^^/^^−^* spermatocytes are shown in (**D**).

Curiously, IHO1 was present on axes in nearly all (97%) late zygotene-early pachytene spermatocytes in the DSB-defective *Spo11^−^^/^^−^* mice (Figure 5A and B). IHO1 also persisted on axes in mice that expressed a catalytically inactive form of SPO11 (*Spo11^YF/YF^*) (30) and mice that lacked DSBs due to the disruption of the SPO11-auxiliary protein MEI4 (Figure 5A and B) (55). These observations suggest that DSBs, and not SPO11 itself, are required for IHO1 depletion from all axes in an early pachytene-equivalent stage. Pre-DSB recombinosome and IHO1 presence on axes correlated in both SC- and DSB-defective spermatocytes, which indicates that the persistence of IHO1 permits the persistence of the full DSB machinery on axes in the absence of DSBs (Supplementary Figure S4).

Most DSBs depend on the β isoform of SPO11, but a second isoform, SPO11α, is required for a late wave of DSBs that enables efficient pairing between the pseudoautosomal regions (PARs) of sex chromosomes in spermatocytes at the zygotene to pachytene transition (31,56). Intriguingly, all-axis depletion of IHO1 in early pachytene follows soon after the formation of SPO11α-dependent late DSBs in wild type.

Hence, we tested if all-axis depletion of IHO1 is triggered specifically by DSBs that depend on SPO11α. To this end, we examined IHO1 dynamics in *Spo11^−^^/^^−^* mice that express the β form of SPO11 from a transgene, hereafter called SPO11 β-only (mice described in (31)) (Supplementary Figure S5). Unlike in *Spo11^−^^/^^−^* spermatocytes, autosomes efficiently synapsed and IHO1 was efficiently depleted from unsynapsed sex chromosomes in SPO11β-only spermatocytes in early pachytene (Supplementary Figure S5B). Thus, IHO1 depletion in early pachytene does not require the α form of SPO11 or a late wave of DSB formation.

To address if all-axis IHO1 depletion required SPO11-dependent DSBs, we tested if γ-irradiation-induced DSBs supported IHO1 depletion in a *Spo11^−^^/^^−^* background. Hence, we detected IHO1 in *in vitro* cultures of *Spo11^−^^/^^−^* spermatocytes two and four days after 4 Gray of γ-irradiation (Figure 5C). In these experiments, we scored the fraction of cells where IHO1 was completely depleted from all axes, which contrasted the earlier described irradiation experiments where axial distribution of IHO1 was examined in IHO1-positive cells (Figure 1D). Interestingly, IHO1 was depleted from significantly higher fractions of *Spo11^−^^/^^−^* spermatocytes as compared to unirradiated controls, indicating that both SPO11-dependent and -independent DSBs enable all-axis depletion of IHO1 from unsynapsed chromosome axes in an early pachytene-equivalent prophase stage (Figure 5D).

### DNA damage response signaling is required for efficient IHO1 depletion from all axes

**Figure 6.**
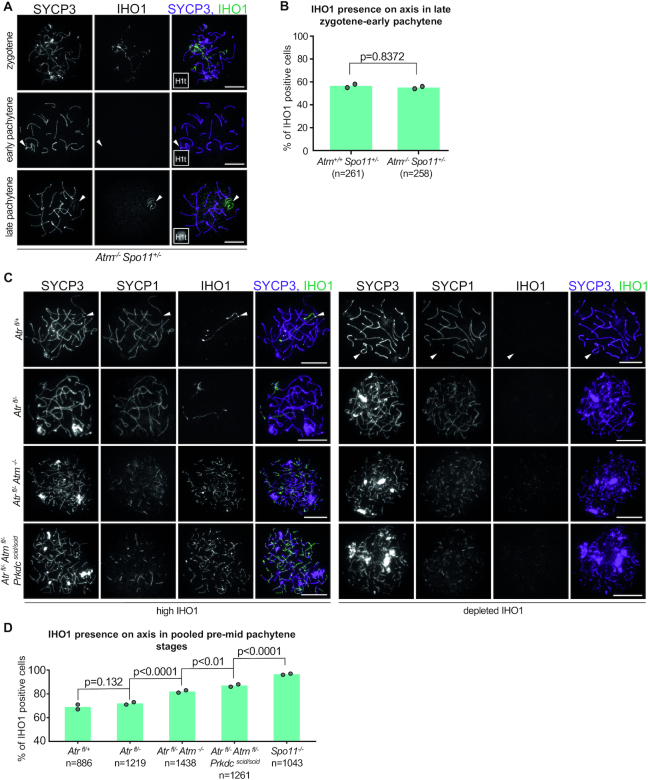
Combined effect of DDR PIKKs determines all axis IHO1 depletion. (**A, C**) Spermatocyte spreads in which we immunostained IHO1, the chromosome axis (SYCP3) and either a synapsis marker (SYCP1, **C**) or a prophase stage marker that is expressed only after early pachytene (histone H1t, (**A**) miniaturized images). (**A**) Sex chromosomes are marked by arrowheads in pachytene *Atm^−^^/^^−^ Spo11^+/^^−^* spermatocytes. For comparison with wild type, see Figure 3D and prior publication (16). (**C**) Late zygotene (left panel top) and early pachytene (right panel top) *Atr^fl^^/+^* spermatocytes are shown together with zygotene-like *Atr^fl^^/^^−^*, *Atr^fl^^/^^−^Atm^−^^/^^−^*, *Atr^fl^^/-^ Atm^fl^^/-^ Prkdc^scid^^/^^scid^* spermatocytes that represent the most advanced axis and SC development stages characteristic of the corresponding genotypes. Arrowheads mark sex chromosomes in *Atr^fl^^/+^* spermatocytes. (**B**) Quantification of IHO1 presence on axes in late zygotene-early pachytene spermatocytes of indicated genotypes. Late zygotene-early pachytene spermatocytes were identified based on their fully developed axes and an absence of the post-early pachytene marker histone H1t. Graph shows the proportion of IHO1 positive spermatocytes. Block bars show averages of two experiments, total numbers of counted cells are indicated. A likelihood ratio test indicated no significant difference between *ATM^−^^/^^-^ Spo11^+/^^−^* and *ATM^+/+^ Spo11^+/^^−^* spermatocytes (*P* = 0.8372). (**D**) The graph shows the proportion of IHO1 positive spermatocytes in pooled pre-mid pachytene stages in the indicated genotypes. Block bars show averages of two experiments, total numbers of counted cells are indicated. Likelihood ratio test was used to calculate the *P* values for the indicated pairwise comparisons between genotypes.

Given that DSBs are required for IHO1 depletion from all axes in early pachytene, we set out to test if the three DDR PIKKs – ATM, ATR and PRKDC – played a role. We examined the effect of ATM-deficiency on IHO1 dynamics in a *Spo11^+/^^−^* background to mitigate the confounding effect of excessive DSBs in the absence of ATM. Loss of ATM did not significantly alter the frequency of all-axis IHO1 depletion in late zygotene-early pachytene spermatocyte populations (Figure 6A and B). Loss of ATR leads to a mid pachytene arrest (57), but a fragmentation of chromosome axes in a considerable proportions of spermatocytes prevents unambiguous identification of late zygotene-early pachytene cells based on chromosome axis morphology (40). Hence, we quantified the proportion of IHO1-negative spermatocytes in pooled pre-mid pachytene stages to compare all-axis depletion of IHO1 in ATR-proficient and -deficient spermatocytes (Figure 6C). Disruption of both ATR and ATM, but not ATR alone, resulted in a significant decrease in the proportions of IHO1-negative pre-mid pachytene spermatocytes as compared to ATR- and ATM-proficient controls (Figure 6D). The disruptive *scid* mutation of PRKDC further decreased the proportions of IHO1-negative spermatocytes in pre-mid pachytene stages in an ATR-ATM-double-deficient background. Thus, ATM, ATR and PRKDC seem to play redundant roles in enabling the depletion of IHO1 from all axes in response to DSBs in early pachytene. Curiously, IHO1 depletion was observed in significantly higher proportions of ATR-ATM-PRKDC-triple-deficient spermatocytes than *Spo11^−^^/^^−^* spermatocytes. This difference may indicate that DSBs enable IHO1 depletion also independent of ATM, ATR and PRKDC by an unknown mechanism. We add the caveat, however, that only conditional disruption of *Atm* and *Atr* was possible in the ATR-ATM-PRKDC-triple-deficient lines because of embryonic lethality when *Atm*^−/−^ and *Prkdc^scid^^/^^scid^* genotypes are combined (40). Due to the conditional gene disruption, it is likely that low levels of residual DDR PIKK activity were still present in ATR-ATM-PRKDC-triple-deficient spermatocytes (40), which may account for the difference from *Spo11^−^^/^^−^*. Hence, it is reasonable to speculate that ATM, ATR and PRKDC are the main if not the only mediators of the DSB-dependent signaling that enables depletion of IHO1 from all axes in early pachytene.

### IHO1 re-accumulation on unsynapsed sex chromosomes and desynapsing autosomes is dispensable for gametogenesis

IHO1 disappearance from all chromosomes in early pachytene may reflect a need to terminate DSB activity to prevent unnecessary DNA damage once all autosomes are synapsed in pachytene. Curiously, in wild type, IHO1 reappears on both unsynapsed sex chromosomes and de-synapsing autosome axes in mid-late pachytene and diplotene, respectively (16). Whereas IHO1 is not expected to promote DSBs after mid pachytene, IHO1 accumulation on unsynapsed axes may reflect an unexpected IHO1 function in late pachytene and diplotene. Alternatively, pachytene–diplotene dynamics of IHO1 may reflect a need to actively suppress IHO1 in early pachytene to terminate DSB formation, and a relaxed control of IHO1 expression and localization after mid pachytene, where IHO1 may be neutral to recombination due to the absence of other SPO11 auxiliary proteins (55).

**Figure 7.**
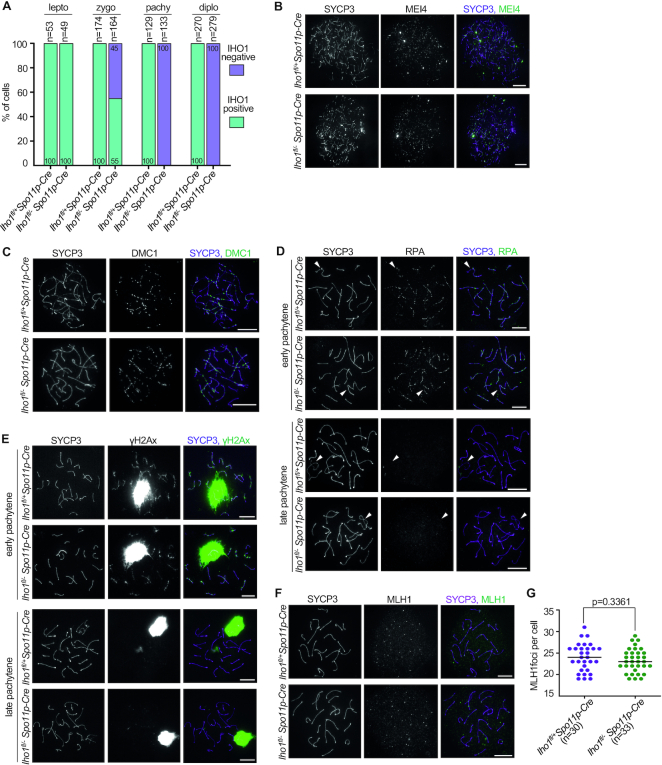
IHO1 loss after zygotene does not disrupt meiotic recombination. (**A**) Quantification of IHO1 presence on axes in spermatocytes of *Iho1^fl/+^Spo11p-Cre* and *Iho1^fl/^^−^Spo11p-Cre* mice. IHO1, SYCP3 (axis marker) and histone H1t (stage marker) were detected in spermatocytes of adult mice. Representative images of spermatocytes are shown in Supplementary Figure S6. Graph shows the proportion of IHO1 positive and negative spermatocytes in leptotene, zygotene, mid-late pachytene and diplotene stages. Histone H1t negative early pachytene spermatocytes were excluded from the comparison as IHO1 is depleted from axes in the *Iho1^fl/+^Spo11p-Cre* control samples. Combined datasets from two experiments are shown, total numbers of counted cells are indicated. (**B–F**) Indicated proteins were detected by immunofluorescence in nuclear surface-spread spermatocytes of adult *Iho1^fl/+^Spo11p-Cre* and *Iho1^fl/^^−^Spo11p-Cre* mice. Spermatocytes are shown in (**B**) leptotene, (**C**) late zygotene, (**F**) late pachytene or (**D, E**) in the indicated prophase stages. Arrowhead marks sex chromosomes in (**D**). Bars, 10 μm. (**G**) Quantification of axis associated MLH1 focus in late pachytene spermatocytes. Numbers of analyzed cells (*n*) and medians (bars) are indicated. Median focus numbers were 24 and 23 for *Iho1^fl/+^Spo11p-Cre* and *Iho1^fl/^^−^Spo11p-Cre*, respectively. Mann–Whitney *U* test indicated no significant difference.

To distinguish between these possibilities, we set out to inactivate IHO1 after it performed its essential function in DSB formation. We conditionally disrupted IHO1 by combining a floxed allele of *Iho1* (*Iho1^fl^* (16)) with a previously described *Spo11p-Cre* transgene (35), which expresses Cre recombinase under the control of the *Spo11* promoter (see Supplementary Figure S6A for explanation of IHO1 alleles). SPO11 is expressed from early prophase but its expression greatly increases in early pachytene (58). Therefore, we postulated that the *Spo11p-Cre* transgene was potentially suitable for IHO1 inactivation after IHO1 performed its function in DSB formation but before IHO1 re-accumulated in late pachytene. We tested this idea by monitoring IHO1 localization in chromosome spreads of *Iho1^fl/^^−^Spo11p-Cre* and, as a control, *Iho1^fl/+^Spo11p-Cre* spermatocytes (Figure 7 and Supplementary Figure S6).

Whereas IHO1 was present on chromosome axes in all leptotene and the majority of zygotene (55%, *n* = 164 cells) spermatocytes, IHO1 was never detected on axes beyond mid pachytene in *Iho1^fl/^^−^Spo11p-Cre* mice (Figure 7A and representative images in Supplementary Figure S6). In contrast, IHO1 was present on unsynapsed axes in all control *Iho1^fl/+^Spo11p-Cre* spermatocytes in leptotene to zygotene and late pachytene to diplotene stages. Thus, IHO1 was efficiently depleted beyond zygotene in *Iho1^fl/^^−^Spo11p-Cre* mice. *Iho1^fl/^^−^Spo11p-Cre* spermatocytes proficiently formed foci of pre-DSB recombinosomes (MEI4) and DSB markers (DMC1 and RPA) (at least 50 cells inspected for each relevant prophase stage, Figure 7B–D). Further, chromosomes efficiently paired and synapsed upon progression to pachytene (Supplementary Figure S6B-G). These observations suggest that IHO1 function is mostly preserved in DSB formation in *Iho1^fl/^^−^Spo11p-Cre* mice, which is consistent with the observation that IHO1 depletion started only in zygotene and affected only a minority of zygotene spermatocytes,

To test if post-zygotene depletion of IHO1 caused an impairment in meiotic recombination, we monitored unrepaired DSB markers (RPA foci and autosomal flares of γH2AX, Figure 7D and E) and a crossover marker (MLH1 foci, Figure 7F) as *Iho1^fl/^^−^Spo11p-Cre* spermatocytes progressed in pachytene. Whereas γH2AX that marked unsynapsed chromatin on sex chromosomes persisted, RPA foci (median 2.5 foci in late pachytene, n = 13 for both genotypes) and autosomal flares of γH2AX disappeared after spermatocytes transitioned from early to late pachytene in both *Iho1^fl/+^Spo11p-Cre* and *Iho1^fl/^^−^Spo11p-Cre* mice. Thus, the repair of DSBs appears efficient in *Iho1^fl/^^−^Spo11p-Cre* mice. MLH1 focus numbers were also similar in *Iho1^fl/+^Spo11p-Cre* and *Iho1^fl/^^−^Spo11p-Cre* mice, suggesting that IHO1 presence in pachytene is dispensable for crossover formation (Figure 7F and G).

The distantly related orthologue of IHO1 in *Sordaria*, Mer2, associates with chromosomes and promotes chromosome compaction in diplotene (59). We found no obvious change in chromosome axis length in *Iho1^fl/^^−^Spo11p-Cre* spermatocytes as compared to *Iho1^fl/+^Spo11p-Cre* spermatocytes, indicating that the late prophase Mer2 function in chromosome compaction is not conserved between *Sordaria* Mer2 and mammalian IHO1. Consistent with these observations, *Iho1^fl/^^−^Spo11p-Cre* mice were fertile (average litter sizes were 18 and 16.67 pups in litters of four *Iho1^fl/^^−^Spo11p-Cre* and three control mice, *P* = 0.3548 as calculated by unpaired t-test) and showed no obvious abnormality in testis cellularity (Supplementary Figure S7). Thus, *Iho1^fl/^^−^Spo11p-Cre* phenotypes strongly suggest that IHO1 does not serve a critical role in late prophase, hence accumulation of IHO1 on unsynapsed axes after mid pachytene may merely reflect relaxed control of IHO1 expression.

## DISCUSSION

**Figure 8.**
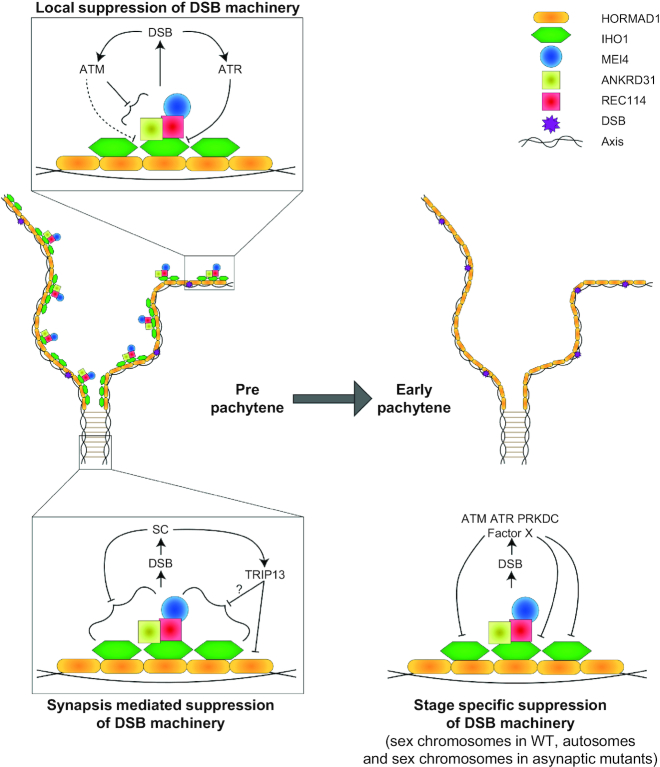
Model for DSBs-promoted disruption of the DSB machinery. Graphical representation of DSB-promoted feedback mechanisms that suppress the DSB machinery in meiocytes. Arrows and the blocking arrows indicate promotion and inhibition, respectively. Grey block arrow represents cell cycle progression. Schematic models of the DSB machinery is shown on partially synapsed chromosome axes in pre-pachytene and early pachytene stages (middle), and the three boxed models (top and bottom) show three distinct types of regulatory interactions between DSBs and the DSB machinery. We hypothesize that pre-DSB recombinosomes enable DSB formation on axis from leptotene onset. (top left model) Shortly after their initiation, DSBs activate ATM leading to a suppression of pre-DSB recombinosomes, which restricts further DSB formation. Single-stranded DNA ends that result from processing of DSBs activates ATR, and ATR promotes depletion of IHO1 from axes in the vicinity of DBSs. This local IHO1 depletion may prevent future assembly of pre-DSB recombinosomes adjacent to DSBs, which may help to distribute DSBs along chromosome axes. (bottom left model) DSB initiated recombination promotes synapsis, and synapsis restricts the DSB machinery to asynapsed regions, where further DSBs are useful for promoting SC formation. (bottom right model) Finally, DSBs enable an enigmatic timing mechanism that depletes the DSB machinery from unsynapsed axes by the time of pachytene onset, where wild-type meiocytes completes synapsis between homologous chromosomes. This mechanism may protect the sex chromosomal genome as XY chromosomes are only partially synapsed in pachytene in wild type.

We report on four negative feedback mechanisms that deplete complexes of SPO11 auxiliary proteins from chromosomes in response to DSBs (Figure 8). First, DSBs activate an ATM dependent mechanism that limits pre-DSB recombinosome numbers without disrupting the IHO1 platform on which pre-DSB recombinosomes assemble. Second, in their vicinity, DSBs trigger ‘local’ depletion of IHO1 from unsynapsed axes. Third, DSBs enable homologue synapsis, and the resultant SCs promote IHO1 depletion from synapsed axes. Fourth, DSBs are necessary for a mechanism that depletes IHO1 from all axes, including unsynapsed axes, at the onset of pachytene. Together these mechanisms help to restrict assembly of the DSB machinery to chromatin regions and prophase stages where DSBs are functional for promoting homologue pairing. Hence, we propose that these mechanisms maximize the utility of DSBs for homology search while limiting their potential genotoxicity.

### DSBs trigger local disruption of the DSB machinery by distinct ATM- and ATR-dependent mechanisms

One of the key challenges of meiotic initiation is to prevent the DSB machinery from activating SPO11 multiple times in the same chromosomal locations. Continuous or repeated SPO11 activity in the same axis regions is expected to cause adjacent DSBs either within the same DNA molecules or within sister chromatids that share axis in each homologue. We speculate that, if per-cell DSB numbers are kept equal, clustered DSBs are less efficient than evenly distributed DSBs in ensuring SC formation along the entire lengths of chromosomes. Correspondingly, it is likely that repeated DSB formation in the same chromosomal regions increases the burden on the DSB repair machinery without proportional benefits for SC formation. Further, breakage of both sister chromatids in the same regions may also cause chromosome fragmentation, and if pairs of sister chromatids are broken in both homologues in the same chromosomal positions then DSBs are not reparable by template-guided recombination. Given these considerations, it is expected that the DSB machinery is locally disabled once it has successfully promoted DSB formation.

We discovered a negative correlation between the spatial distributions of DSB foci and IHO1 on chromosome axes, which may reflect a local disruption of the DSB machinery following DSB formation. We also considered preferential formation of DSBs or preferential binding of DSB-generated DNA ends in regions where IHO1 levels are low as further potential mechanisms underlying the negative correlation between DSB foci and IHO1. However, we disfavor the idea of DSB formation in low IHO1 regions because it is contrary to current models of DSB formation and the essential role of IHO1 in promoting DSBs. Our observations also argue against differential recruitment of DSBs to IHO1–negative regions, because repair foci of irradiation-induced DSBs, which are randomly placed in the genome, do not correlate negatively with IHO1 distribution initially. Interestingly, negative correlation emerges between irradiation-induced repair foci and IHO1 with a time delay. A straightforward interpretation is that unrepaired DSBs promote IHO1 depletion in their vicinity as part of a negative feedback mechanism that disables repeated DSB formation in adjacent sites.

Observations in prior reports suggested that a DDR PIKK, ATM, signals from newly formed DSBs to stop further DSB formation. Specifically, ATM appears to suppress bulk SPO11 activity in a wide variety of taxa including budding yeast, Drosophila and mice (21–25,27). Further, the genome-wide distribution of DSBs in the absence of ATM is consistent with clustered DSB formation (24,28), suggesting an ATM role in suppressing SPO11 activity adjacent to DSBs. In contrast to ATM, ATR does not limit total DSB numbers (40,51). The third main DDR PIKK, PRKDC, is thought to play only a minor role in meiotic recombination, and has not been reported to function in DSB formation (38). Thus, prior observations predicted that the DSB machinery is suppressed primarily by ATM in response to DSBs.

Surprisingly, we found that ATR deficiency leads to a loss of negative correlation between the positions of IHO1 and DMC1/RAD51 foci on axis. Whereas ATR loss decreases RAD51/DMC1 focus numbers (40,51), we consider it unlikely that a potentially altered DSB activity or altered RAD51/DMC1 focus positioning causes loss of negative correlation between IHO1 and RAD51/DMC1 foci. There are two arguments: first, ATR-deficient spermatocytes seem to be proficient in forming DSBs as measured by SPO11-oligo levels (40,51), second, neither decreasing DSB levels (*Spo11^+/^^−^*) nor increasing DSB activity (*Atm^−^^/^^−^ Spo11^+/^^−^*) nor randomizing distribution of DSBs (irradiation induced DSBs in *Spo11^−^^/^^−^* meiocytes) result in loss of negative correlation between IHO1 and DMC1/RAD51. Therefore, we favor the hypothesis that ATR enables IHO1 depletion in the vicinity of DSBs. ATM seems to play only a small secondary role in IHO1 regulation, and this role is detectable only in the absence of ATR. In contrast, absence of ATM, and not the absence of ATR, results in a strongly elevated density of pre-DSB recombinosomes on unsynapsed axes. ATM may destabilize pre-DSB recombinosomes in the vicinity of DSBs, or, alternatively, ATM may inhibit pre-DSB recombinosome assembly and/or maintenance not only locally but across the nucleus. Regardless, ATM but not ATR limits bulk pre-DSB recombinosome numbers. These observations suggest that DSBs limit the formation and/or stability of pre-DSB recombinosomes by both ATM- and ATR-dependent mechanisms, but with distinct consequences for DSB formation.

ATM is activated by blunt ended or shortly resected DNA ends shortly after DSB formation. In contrast, ATR activation requires single-stranded DNA ends that emerge only later due to ATM-promoted DNA-end resection (60). Hence, we speculate that ATM-mediated suppression of pre-DSB recombinosomes may be an early response to DSBs, which helps to prevent excessive DSB formation and, in particular, the catalysis of repeated and/or clustered DSBs by the same pre-DSB recombinosomes. However, the ATM-mediated mechanism may be insufficient to prevent reassembly of pre-DSB recombinosomes in the vicinity of DSBs, after DSB-generated DNA ends are processed for recombination. Once DNA-end resection generates single-stranded DNA ends, ATM signaling ceases, and ATR signaling takes over (60). ATR may maintain a long lasting suppression of the DSB machinery in the vicinity of DSBs by locally disrupting the IHO1 platform on which pre-DSB recombinosomes assemble. Whereas this ATR-dependent feedback mechanism is expected to restrict formation of new pre-DSB recombinosomes to axis regions that are devoid of DSBs, it does not seem to affect the assembly and disassembly rates of pre-DSB recombinosomes, as density of pre-DSB recombinosomes is similar in the presence and absence of ATR. Hence, we hypothesize that ATR signaling inhibits clustered DSB formation and promotes even distribution of DSBs on unsynapsed axes without restricting the total number of DSBs.

### Synapsis suppresses the DSB machinery by both TRIP13-dependent and independent mechanisms

DSBs are not only suppressed in the vicinity of previously formed DSBs but also in chromosomal regions where homologues stably paired and synapsed. Suppression of DSBs in synapsed regions is accompanied by, and probably requires, the depletion of pre-DSB recombinosome components from chromosome axes. Whereas DDR PIKKs were not required for IHO1 depletion from synapsed axes, the TRIP13 AAA+ ATPase was required. TRIP13 remodeling promotes the removal of HORMAD1 from synapsed axes, and HORMAD1 is a key anchor of IHO1 on axes. Hence, it is likely that TRIP13 enables efficient depletion of IHO1 and pre-DSB recombinosomes by remodeling HORMAD1.

However, TRIP13-mediated HORMAD1 remodeling is not the only mechanism by which synapsis controls DSBs. Albeit with reduced efficiency, MEI4-REC114-IHO1-containing pre-DSB recombinosomes still form in the absence of HORMAD1. The HORMAD1-independent pre-DSB recombinosomes also disappear from synapsed regions revealing a HORMAD1-independent mechanism that disrupts the DSB machinery in synapsed regions (16).

Whereas the HORMAD1-independent suppression of DSBs may involve a possible remodeling of pre-DSB recombinosome components by TRIP13, we favor the involvement of a TRIP13-independent mechanism. Albeit with lower efficiency than in wild-type cells, IHO1 levels are moderately lowered and pre-DSB recombinosomes are disrupted along synapsed axes in TRIP13-deficient cells. These observations suggest that the SC promotes axial depletion of IHO1 and pre-DSB recombinosomes by an as yet uncharacterized TRIP13-independent mechanism next to the TRIP13-mediated remodeling of HORMAD1. Despite this redundancy, TRIP13-dependent depletion of pre-DSB recombinosomes may be critical for DSB suppression in synapsed regions, as early recombination intermediates, which may result from inappropriately persisting DSB-activity, were observed on synapsed axes in TRIP13-deficient spermatocytes (34,61).

### DSBs enable stage-dependent impairment of DSB machinery

Prophase stages that are permissive for pre-DSB recombinosome assembly and DSB formation are characterized by IHO1 presence on chromosome axes. In spermatocytes, IHO1 and pre-DSB recombinosomes are depleted not only from synapsed autosomes but also from the unsynapsed axes of heterologous sex chromosomes at the onset of pachytene. Thus, SPO11 activity is expected to cease on all axes including unsynapsed sex chromosomes after all homologues synapsed. We hypothesize that if DSB activity was sustained on unsynapsed sex chromosomes beyond zygotene, it could lead to increased mutation load in sex chromosome-linked genes and/or meiotic arrest due to DNA-damage checkpoint activation. Hence, a shutdown of the DSB machinery on unsynapsed axes at the onset of pachytene may confer benefits in the heterogametic sex of organisms that have heterologous sex chromosomes.

Interestingly, whereas IHO1 disappears from chromatin at the onset of pachytene, MEI4, REC114 and ANKRD31 persist in aggregates in PARs of sex chromosomes and PAR-like regions of three autosomes well into, but not beyond, early pachytene (15–17,20). Thus, IHO1 depletion seems to precede the depletion of other pre-DSB recombinosome components, suggesting that IHO1 depletion may be a rate-limiting step in the termination of DSB formation in pachytene. Notably, IHO1, but not other pre-DSB components, reaccumulate on unsynapsed chromosomes beyond mid pachytene, but we did not detect a function for the late wave of IHO1. Hence, we speculate that axial IHO1 reaccumulation may merely reflect relaxed control of IHO1 expression, stability and/or localization once loss of other pre-DSB recombinosome components redundantly disabled the DSB machinery. The mechanism of IHO1 dynamics in pachytene is not known. IHO1 may only transiently lose its association with the axis in early pachytene, or IHO1 may be transiently degraded requiring *de novo* synthesis of IHO1 from mid pachytene.

Whereas the molecular mechanism of IHO1 depletion remains unclear, our analysis revealed the relationship between IHO1 depletion in pachytene and preceding key events in recombination. Importantly, we found that the stage-dependent depletion of IHO1 from all axes did not require the completion of synapsis between homologues in spermatocytes. This observation highlights important properties of the zygotene-pachytene transition. According to the standard definition of prophase stages, late zygotene and early pachytene are stages where axes are fully formed, and synapsis is either incomplete or complete between homologues, respectively. It was, however, poorly understood if the zygotene-pachytene transition represented a significant change in the cellular state of meiocytes beyond the status of the SC. Depletion of IHO1 at the onset of pachytene suggests that the zygotene-pachytene transition is also marked by a loss of DSB competence in wild type. Importantly, a similar timing of IHO1 depletion in wild type and diverse synapsis-defective mutants strongly suggests that the loss of DSB competence and the underlying transition between zygotene-like and pachytene-like cellular states are independent of SC completion. Thus, it is reasonable to conclude that a pachytene-like cellular state exists in SC and DSB repair-defective mutants even if late zygotene and early pachytene stages cannot be distinguished based on axis and SC morphology in these mutants.

Thus, while SC completion and the zygotene-pachytene transition co-occur in wild type, there seems to be no mechanistic or checkpoint-based coupling between SC formation and zygotene-pachytene transition, as detected by IHO1 depletion. By way of exclusion, we conclude that, surprisingly, an unknown timing mechanism ensures that DSBs are suppressed at the same time as SCs are completed at pachytene onset in wild type.

Unexpectedly, we discovered that stage-dependent loss of IHO1 and pre-DSB recombinosomes depended on DSB formation and the activity of all three DDR PIKKs. Thus, DSBs activate a previously unanticipated feedback mechanism to suppress DSB activity genome-wide at the onset of pachytene, where meiocytes are expected to complete SC formation. DSB formation may be required not only for stage-dependent loss of DSB competence, but also for other, as yet unidentified features of the zygotene-pachytene transition. Thus, meiocytes may fail to progress beyond a zygotene-like cellular state in the absence of DSBs. Regardless, both of these scenarios invoke a checkpoint-like regulatory circuit that makes the pachytene-associated shutdown of DSBs dependent on prior recombination initiation in meiocytes.

Notably, this regulatory circuit also effects a temporal separation between DSB formation and the quality control of DSB repair and homologue synapsis. Whereas DSB formation is terminated at pachytene onset, unrepaired DSBs and asynapsis block prophase progression only in mid pachytene. Thus, there is time for the last DSBs to promote completion of synapsis in late synapsing regions before the mid pachytene checkpoint eliminates asynaptic spermatocytes. Further, the time between zygotene and mid pachytene is likely sufficient for the processing of the last DSBs, thereby preventing newly-forming DSBs from triggering a spermatogenic block in mid pachytene.

## DATA AVAILABILITY

The data supporting the findings of this study are available within the paper. The source data underlying both main and supplementary figures are provided as a Source Data file.

## Supplementary Material

gkab082_Supplemental_Files

## Notes

Present address: Marcello Stanzione, Massachusetts General Hospital Cancer Center, Laboratory of Molecular Oncology, Harvard Medical School, Charlestown, MA 02129, USA.
