# Supplementary material for: Four-pronged negative feedback of DSB machinery in meiotic DNA-break control in mice
Source: Nucleic Acids Res. 2021 Feb 22;49(5):2609–28. doi: 10.1093/nar/gkab082 (PMC7969012; doi:10.1093/nar/gkab082)
Supplement: gkab082_Supplemental_Files [file gkab082_supplemental_files.zip › RGB value extractor imageJ macro.rtf]

input = getDirectory("Input directory");
setBatchMode(true);
processFolder (input);
function processFolder(input) {
	list = getFileList(input);
	for (i = 0; i < list.length; i++) {
		if(File.isDirectory(input + list[i])) {
			processFolder("" + input + list[i]);
		
		}
		else { 
		open(input+list[i]);
		
		}
		if(input + list[i]==is("composite")){
			
			process();
			
		}
		else { 
		run("Make Composite");
		process();
		
		}
	
	}
}
//get channel intensities
function process(){

var ROIList = getROIList();

Red_array = newArray(0);

Green_array = newArray(0);

ROI_Array = newArray(0);

for (k=0; k<roiManager("count"); k++) {
	
    roiManager("select", k);
     Stack.setChannel(1);
     rd=getProfile();
     Stack.setChannel(2);
     gr=getProfile();


  for (c=0; c<rd.length; c++){
 	setResult("ROI", c, ROIList[k]);
 	  ROI_Array = Array.concat(ROI_Array, getResultString("ROI",c));
 	
 	}
 	
for (a=0; a<rd.length; a++){
      setResult("Red", a, rd[a]);
      Red_array = Array.concat(Red_array, getResult("Red",a));

}
  for (b=0; b<gr.length; b++){
      setResult("Green", b, gr[b]);
      Green_array = Array.concat(Green_array, getResult("Green",b));
}


for(n=0; n<Green_array.length; n++){
  setResult("Green", n, Green_array[n]);
  setResult("Red", n, Red_array[n]);
  setResult("ROI", n, ROI_Array[n]);
}


name = getTitle; 
index = lastIndexOf(name, "."); 
if (index!=-1) name = substring(name, 0, index); 
name = name + ".csv"; 
saveAs("Measurements", input+name);  
} 
close();
run("Clear Results");
 }

function getROIList(){
	run("To ROI Manager");//runs ROI manager and adds already saved ROI to ROI manager
	ROI_array=newArray(roiManager("count"));//generates an array big as ROI count 
	for (k=0; k<roiManager("count"); k++) {//loops throughROIs
		roiManager("select", k);
		ROI_array[k]=call("ij.plugin.frame.RoiManager.getName", k);
		/*
		*assigns names of each ROI to an array defined earlier
		*/
	
	}

return ROI_array;	
}
setBatchMode(false);
