# Supplementary material for: Four-pronged negative feedback of DSB machinery in meiotic DNA-break control in mice
Source: Nucleic Acids Res. 2021 Feb 22;49(5):2609–28. doi: 10.1093/nar/gkab082 (PMC7969012; doi:10.1093/nar/gkab082)
Supplement: gkab082_Supplemental_Files [file gkab082_supplemental_files.zip › Supplementary figures.pdf]

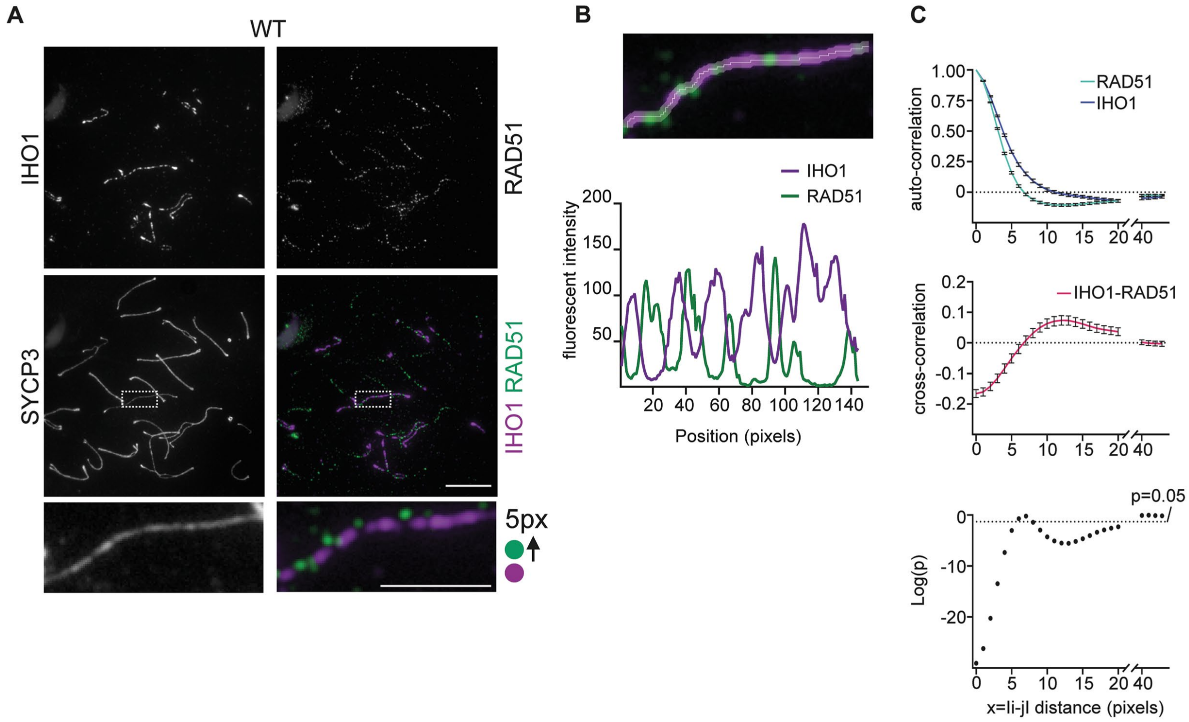

Supplementary Figure 1. Depletion of axial IHO1 in the vicinity of RAD51

(A) IHO1 and markers of the chromosome axis (SYCP3) and unrepaired DSBs (RAD51) were detected in nuclear surface spread spermatocytes of adult wild type by immunofluorescence. Enlarged insets (bottom) show a section of IHO1-rich unsynapsed chromosome axis. RAD51 signal was shifted 5 pixels up in the inset of the overlay image to help comparison between the distribution of RAD51 and IHO1 signals. Bar, 10µm; 5µm in enlarged inset. **(B)** Quantification of axis-associated IHO1 and RAD51 immunofluorescence signal along the axis section that is shown in the enlarged insets in **A**. Signal intensity is shown in arbitrary units. Position is indicated in pixels from left to right along the axis**. (C)** Correlation between IHO1 and RAD51 signals along unsynapsed chromosome axes of wild-type spermatocytes. Correlations were calculated between signals in all positions along the length of axis sections. Top and middle graphs show auto-correlations of IHO1 (blue) and RAD51 (green) signals (top graphs) or cross-correlation between IHO1 and RAD51 signals (middle graphs, red). Averages of correlations are shown for every axial distance between 0 and 20 pixels and between 40 and 43 pixels. x= Ii-jI represents distances between i and j positions along axis sections. Averages of measurements from multiple axis segments of spermatocytes of two mice are shown (see supplementary data file 1 for raw values), error bars indicate standard error of the mean. Bottom graph shows significance of cross-correlation on base 10 logarithmic scale. n=25 cells and n=259 axes were analyzed.

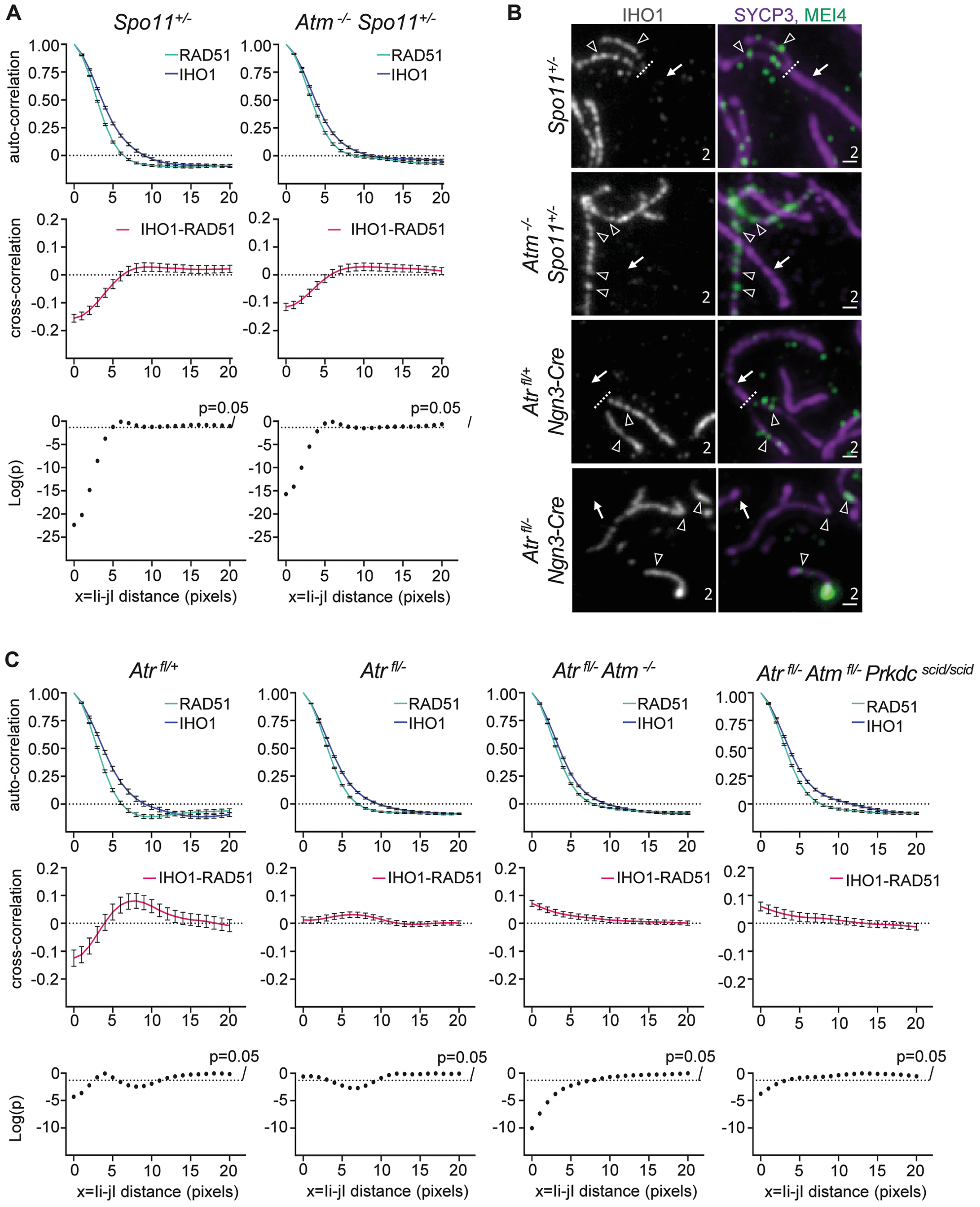

Supplementary Figure 2. Loss of ATM and ATR differentially affects IHO1 depletion from axes in the vicinity of RAD51 marked recombination foci

(A, C) Correlation between IHO1 and RAD51 signals along unsynapsed chromosome axes in spermatocytes of indicated genotypes. Correlations were calculated between signals in all positions along the length of axis sections. Top and middle graphs show auto-correlations of IHO1 (blue) and RAD51 (green) signals (top graphs) or cross-correlation between IHO1 and RAD51 signals (middle graphs, red). Averages of correlations are shown for every axial distance between 0 and 20 pixels, x= Ii-jI represents distances between i and j positions along axis sections, error bars indicate standard error of the mean. Bottom graphs show significance of cross-correlation on base 10 logarithmic scale. Graphs are based on the analyses of n=45 cells and n=178 axes for *Spo11*$^{+/-}$ (**A**), n=46 cells and n=622 axes for *Atm*$^{-/-}$*Spo11*$^{+/-}$ (**A**), n=38 cells and n=514 axes for *Atr*$^{fl/+}$ (**C**), n=64 cells and n=759 axes for *Atr*$^{fl/-}$ (**C**), n=63 cells and n=762 axes for *Atr*$^{fl/-}$*Atm*$^{-/-}$ (**C**), n=40 cells and n=339 axes for *Atr*$^{fl/-}$*Atm*$^{fl/-}$*Prkdc*$^{scid/scid}$ (**C**). (**B**) Enlarged insets (region 2) of spermatocyte images from figure 2B. IHO1 and markers of the chromosome axis (SYCP3) and the pre-DSB recombinosome (MEI4) were detected in spread spermatocytes of indicated genotypes. Synapsed and unsynapsed chromosome regions were identified by the absence or presence of IHO1, respectively. Arrows mark synapsed axes. Triangles mark MEI4 foci on unsynapsed axes. Dotted lines indicate the borders between synapsed and unsynapsed regions of partially synapsed chromosomes in the insets of *Spo11*$^{+/-}$ and *Atr*$^{fl/+}$ *Ngn3-Cre* spermatocytes. Bars, 1µm.

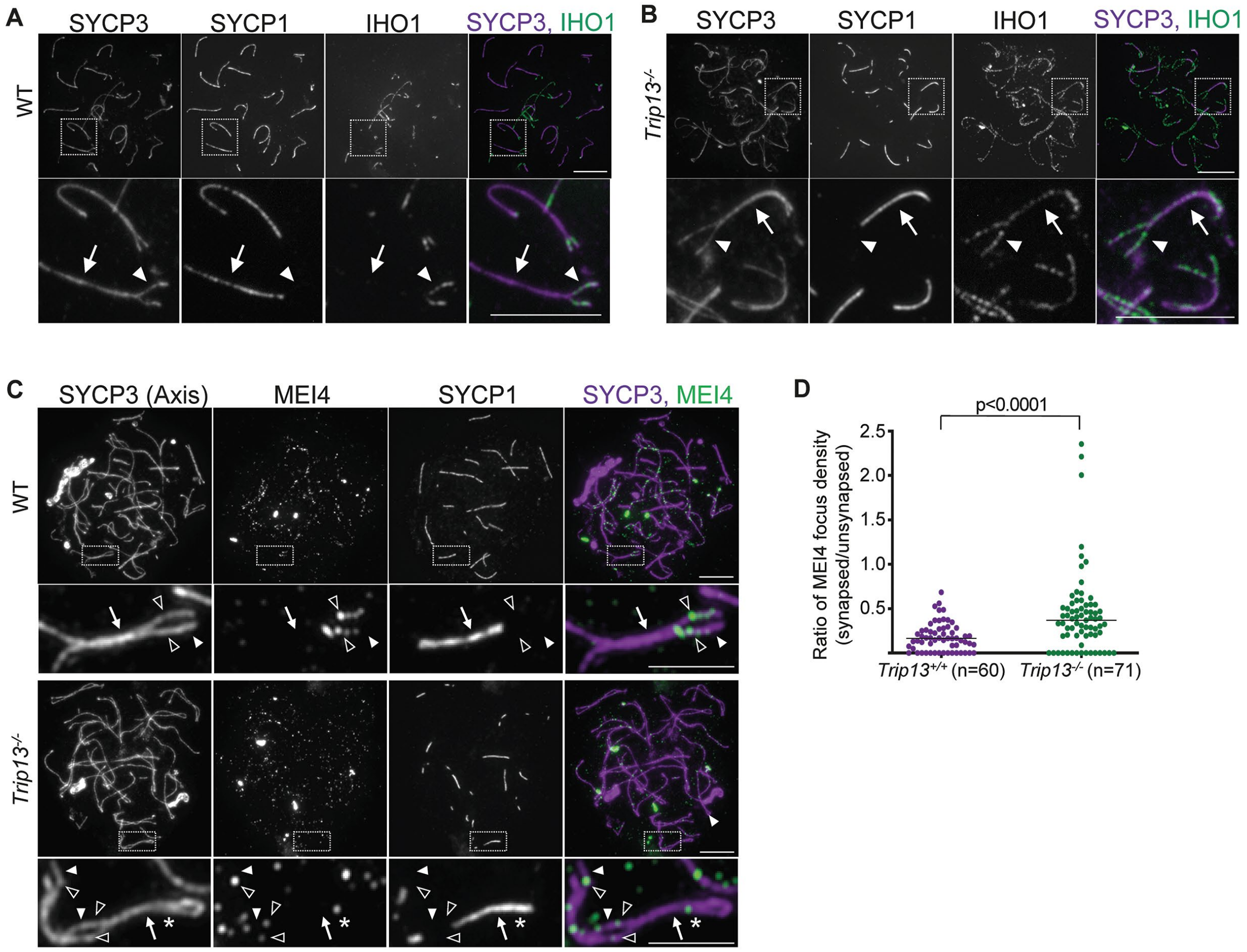

Supplementary Figure 3. MEI4 is depleted from synapsed chromosome axis despite IHO1 persistence in *Trip13*$^{-/-}$ spermatocytes

(**A, B, C**) IHO1 or MEI4 (pre-DSB recombinosome markers), SYCP3 (chromosome axes) and SYCP1 (synapsis marker) were detected in spread spermatocytes of wild-type (**A, C**) or *Trip13*$^{-/-}$ (**B, C**) adult mice by immunofluorescence. Wild-type and *Trip13*$^{-/-}$ spermatocytes are shown in late zygotene or late zygotene-like stages, respectively. (**C**) Enlarged inset show partially synapsed chromosomes. Arrows and arrowheads mark synapsed and unsynapsed axes as identified by the presence or absence of SYCP1 staining, respectively. Triangles and asterisk mark MEI4 foci on unsynapsed and synapsed axes, respectively. Bars, 10µm, insets, 5 µm. (**D**) Graph shows ratio of MEI4 focus densities on unsynapsed axes vs. synapsed axes in late zygotene (wild-type) or late zygotene-like (*Trip13*$^{-/-}$) spermatocytes. Numbers of analyzed chromosomes (n) per category are indicated, data were pooled from two experiments. Results of Mann-Whitney U test are shown.

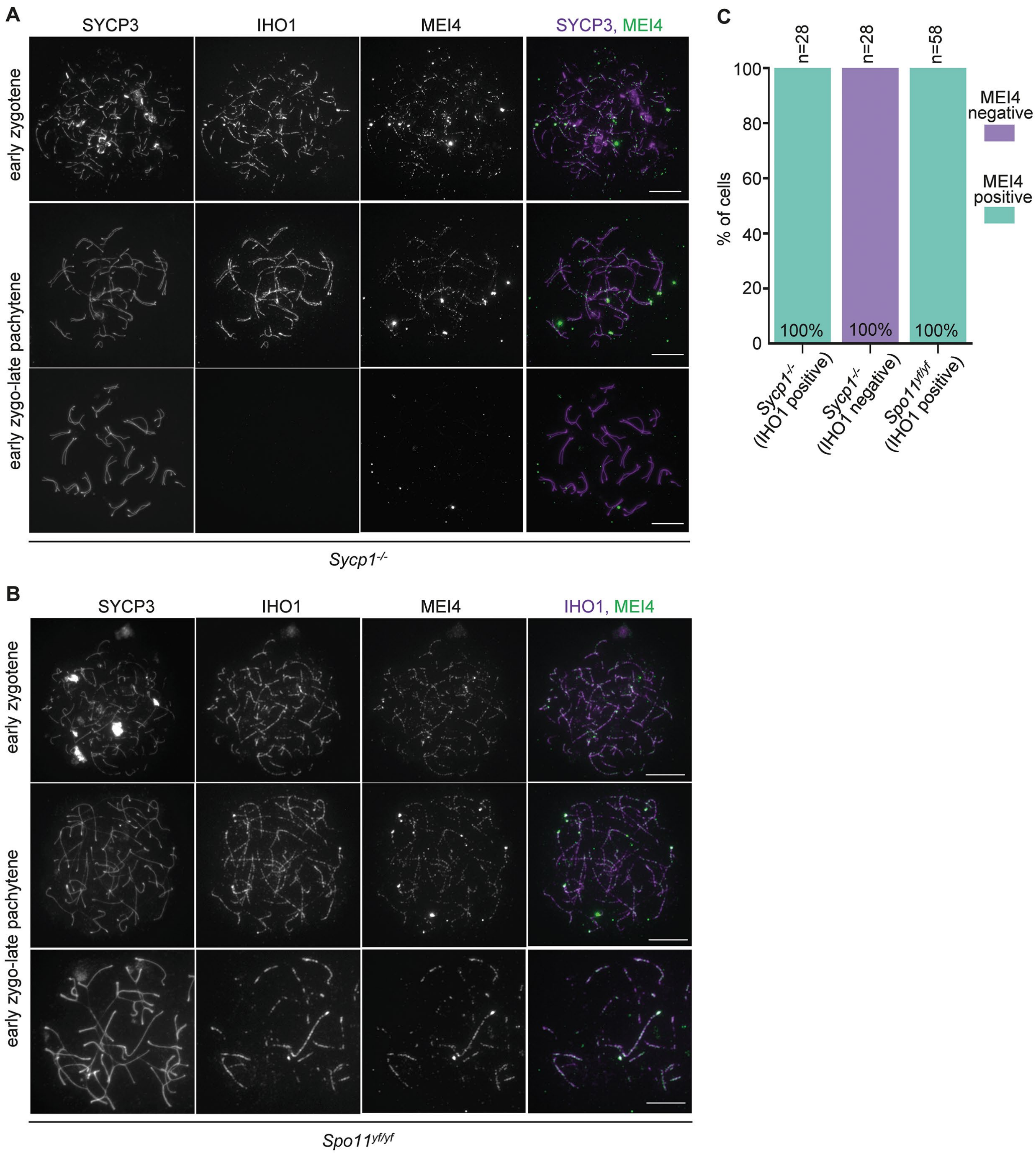

Supplementary Figure 4. Presence of IHO1 and pre-DSB recombinosomes on axes correlate in late zygote-early pachytene stages in SC and DSB defective mutants

(A, B) IHO1, MEI4 (pre-DSB recombinosome marker), and SYCP3 (chromosome axis marker) were detected in spread spermatocytes of **(A)** *Sycp1*$^{-/-}$ or (**B**) *Spo11*$^{YF/YF}$ adult mice in early zygotene and late zygotene- early pachytene stages. The middle and bottom rows show examples of late zygotene-early pachytene spermatocytes where chromosome axes are either frail or well-condensed, respectively. Bars, 10µm. **(C)** Graph shows the percentage of spermatocytes where MEI4 focus was detectable on unsynapsed axes. Numbers of analyzed cells (n) per category is indicated.

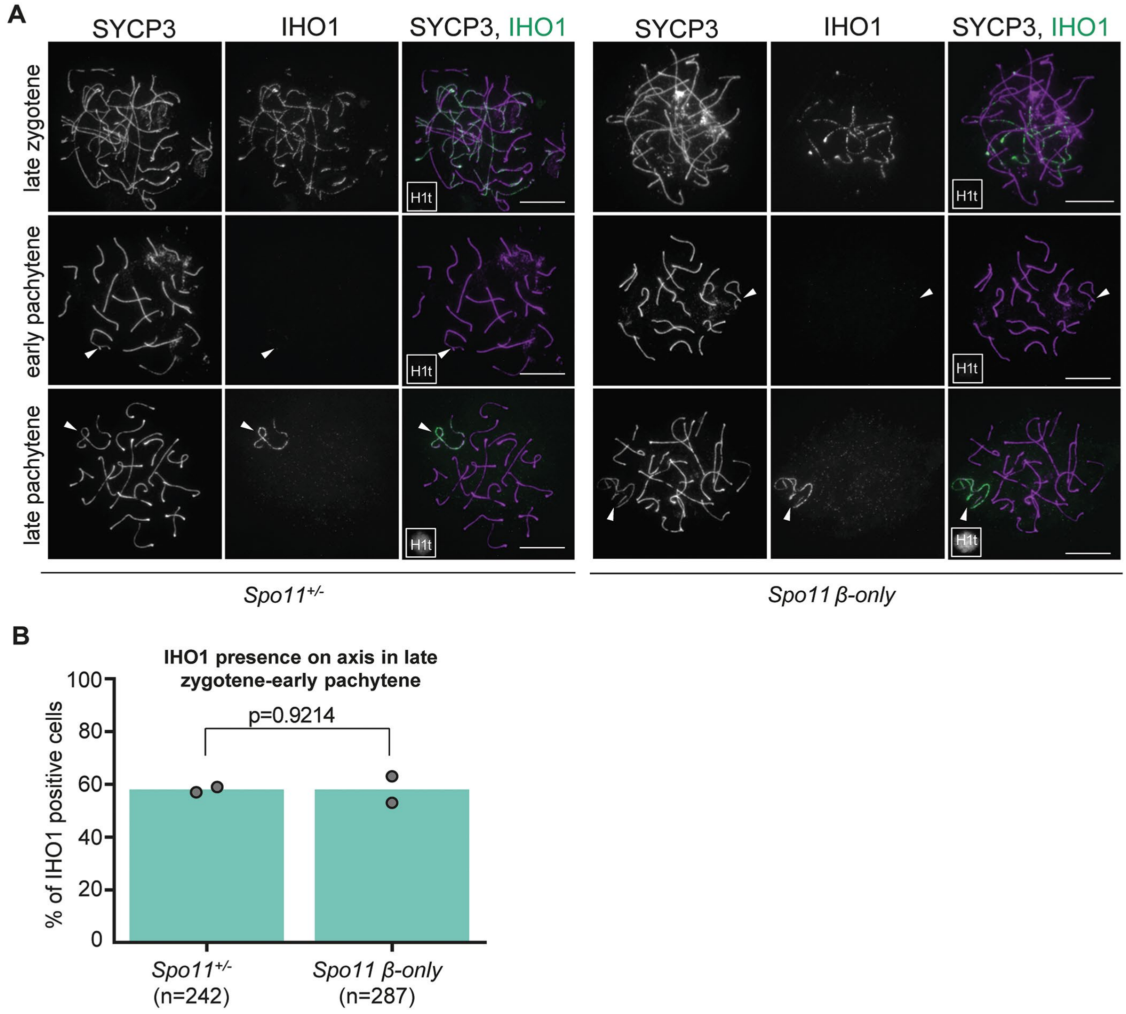

Supplementary Figure 5. IHO1 is depleted from all axes in early pachytene in the absence of the SPO11 α isoform

(A) IHO1**,** SYCP3 (chromosome axis marker) and histone H1t (prophase stage marker, miniaturized images) were detected in spread spermatocytes of *Spo11 β-only* or control *Spo11*$^{+/-}$ adult mice. Late zygotene (upper panel), early pachytene (middle panel) and late pachytene (bottom panel) spermatocytes are shown. Arrowhead marks sex chromosomes. Bars, 10µm. **(B)** Quantification of IHO1 presence on the axes in late zygotene-early pachytene stages in spermatocytes of the indicated genotypes. Late zygotene-early pachytene spermatocytes were identified based on their fully developed axes and an absence of the post-early pachytene marker histone H1t. Graph shows the proportion of IHO1 positive spermatocytes. Block bars show averages of two experiments, total numbers of counted cells are indicated. A likelihood-ratio test indicated no significant difference between *Spo11 β-only* and control *Spo11*$^{+/-}$ spermatocytes (p=0.9214).

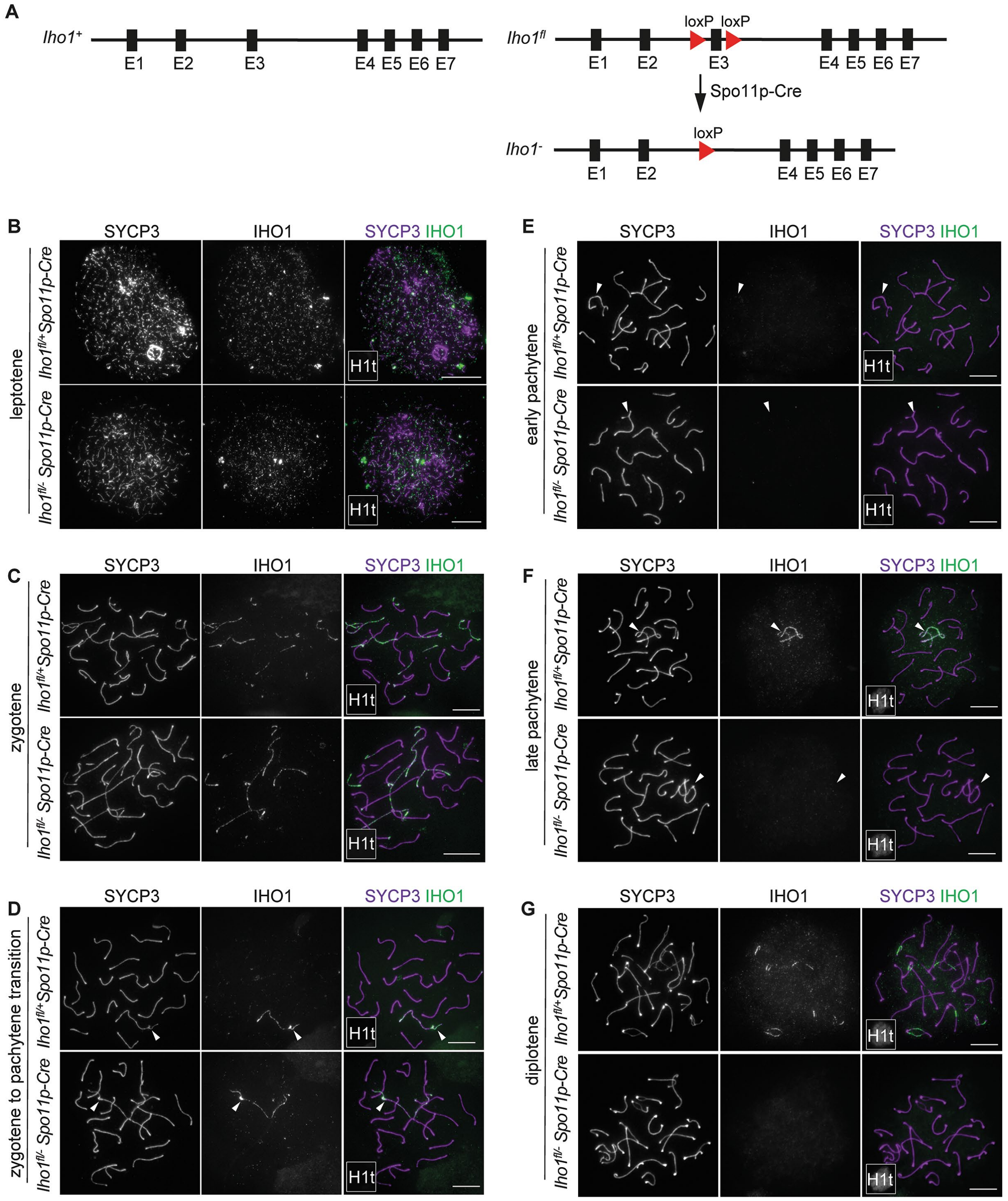

Supplementary Figure 6. Conditional disruption of *Iho1* by *Spo11* promoter-controlled Cre recombinase efficiently depletes IHO1 from post-zygotene spermatocytes

(A) Schematics of the wild-type (*Iho1*$^{+}$) and modified *Iho1* genomic loci. Black boxes represent exons (not to scale). *Iho1*^{fl} is a functional allele where the third exon (E3) is surrounded by loxP sites (red triangles). Cre recombinase under the control of the *Spo11* promoter (Spo11p-Cre) was used to mediate recombination between loxP sites. In the resultant *Iho1*$^{-}$ allele, exon 3 is missing leading to a frameshift after the 80th codon. **(B-G)** IHO1, SYCP3 (axis marker) and histone H1t (miniaturized images) immunofluorescent staining is shown in spread spermatocytes of adult *Iho1*$^{fl/+}$*Spo11p-Cre* and *Iho1*$^{fl/-}$*Spo11p-Cre* mice in the indicated prophase stages. Histone H1t staining was used to identify the meiotic stage in cells with fully formed chromosome axes. Arrowhead marks sex chromosomes. Bars, 10µm

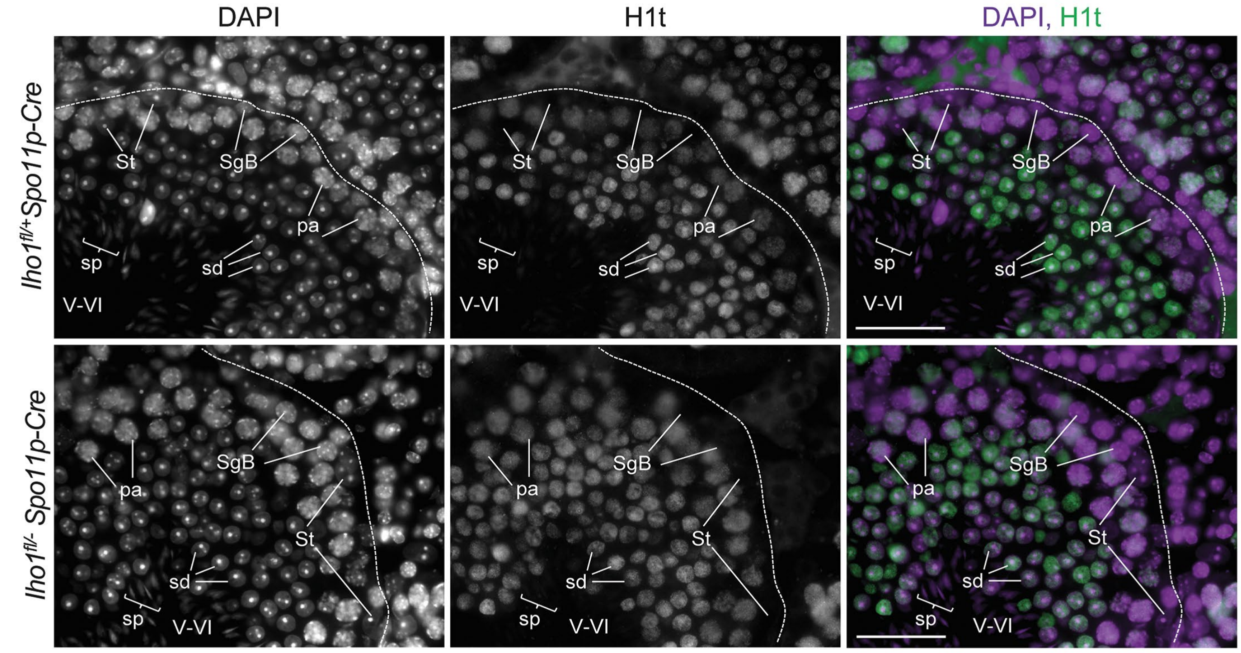

Supplementary Figure 7. Presence of post-meiotic cells in testis of *Iho1$^{fl/-}$Spo11p-Cre* mice

DNA and histone H1t (staging marker of seminiferous tubules) were detected by DAPI and immunostaining, respectively, in sections of testes from adult *Iho1$^{fl/+}$Spo11p-Cre* and *Iho1$^{fl/-}$Spo11p-Cre* mice. Epithelial cycle stages of the seminiferous tubules are indicated by roman numbers. Type B spermatogonia (SgB), pachytene (pa), round spermatids (sd) and sperm (sp) and Sertoli cells (St) are marked. Outlines of tubules (white dashed line). Bars, 50 μm.
